# Allergens abrogate antiinflammatory DNA effects and unmask macrophage-driven neutrophilic asthma via ILC2/STING/TNF-**α** signaling

**DOI:** 10.1172/JCI187907

**Published:** 2025-06-17

**Authors:** Anand Sripada, Divya Verma, Rangati Varma, Kapil Sirohi, Carolyn Kwiat, Mohini Pathria, Mukesh Verma, Anita Sahu, Vamsi Guntur, Laurie Manka, Brian Vestal, Camille Moore, Richard J. Martin, Magdalena M. Gorska, John Cambier, Andrew Getahun, Rafeul Alam

**Affiliations:** 1 Division of Allergy & Immunology,; 2 Division of Pulmonary and Critical Care Medicine, Department of Medicine, National Jewish Health, Denver, Colorado, USA.; 3 Department of Medicine, University of Colorado Denver, School of Medicine, Denver, Colorado, USA.; 4 Department of Immunology & Genomic Medicine, National Jewish Health, Denver, Colorado, USA.; 5 Department of Immunology and Microbiology, University of Colorado Denver, School of Medicine, Denver, Colorado, USA.

**Keywords:** Immunology, Inflammation, Pulmonology, Asthma

## Abstract

The mechanisms of neutrophilic and mixed neutrophilic-eosinophilic asthma are poorly understood. We found that extracellular DNA and nucleosomes (Nucs) were elevated in the airways of patients with neutrophilic-eosinophilic asthma and correlated with bronchoalveolar lavage neutrophils. Bronchial tissue from neutrophilic-eosinophilic asthma had more DNA sensor–positive cells. Intranasally administered DNA did not induce airway hyperreactivity (AHR) or any pathology but induced AHR and neutrophilic-eosinophilic inflammation when coadministered with the allergen *Alternaria* (Alt). Nuc alone induced antiinflammatory/defensive genes, whereas the Nuc-Alt combination increased levels of TNF-α and innate cytokines. The Alt-Nuc phenotype was abolished in *Cgas^–/–^*, *ALR^–/–^*, *Sting^–/–^*, *LysM*Cre:*Sting*^fl/fl^, *IL7R*Cre:*Rorα*^fl/fl^, and *Tnfr2^–/–^* mice. Alt, unexpectedly, played an essential role in the Nuc-induced phenotype. It abrogated Nuc induction of antiinflammatory genes, facilitated Nuc uptake, induced type 2 innate lymphoid cells, which, in the presence of Nuc, produced high levels of TNF-α, and promoted neutrophilic infiltration. We established a paradigm whereby allergens inhibit the antiinflammatory effects of DNA/Nuc and facilitate STING-TNF-α–driven neutrophilic-eosinophilic inflammation in asthma.

## Abstract 2

Inhaled DNA induces anti-inflammatory molecules. Inhaled allergens abrogates this anti-inflammatory property of DNA and co-opt DNA to elicit a form of asthma with mixed neutrophilic-eosinophilic inflammation

## Graphical abstract

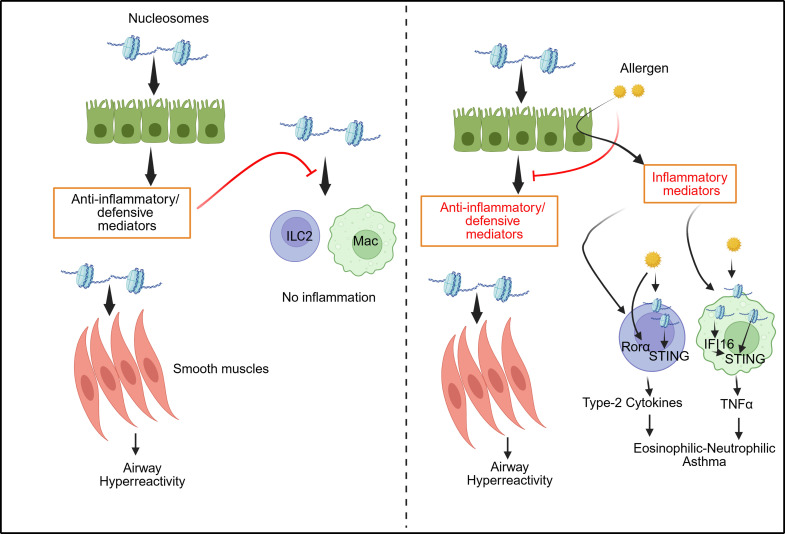

## Introduction

Allergic sensitivity and a type 2 immune response are characteristics of most patients with asthma (1). This is supported by the success of anti–type 2 cytokine and anti-IgE Abs (2). Nonetheless, a substantial fraction of patients does not respond to anti–type 2 modalities (3). This subgroup of patients with asthma has increased neutrophils with or without eosinophils in the airways and are referred to as having neutrophilic asthma (NA). The presence of pathogenic bacterial strains and microbial dysbiosis in the airways of a subgroup of patients with NA and their favorable response to prolonged antibiotic therapy have been reported (4–6). Subclinical chronic infection may explain neutrophilic inflammation in 20%–30% of patients with refractory asthma. The cause of NA in the remainder of patients is unknown. Viral infections trigger asthma exacerbation (7). Whether they cause chronic neutrophilic inflammation is unclear.

DNA is ubiquitously present in the environment, mucosal surface, and body fluids (8). Exogenous DNA (microbial and host-derived), when internalized into the cell, elicits a wide range of biological responses, including apoptosis, anergy, immunosuppression, immunostimulation, inflammation, and autoimmunity (9, 10). Cytosolic and endosomal/lysosomal DNases protect cells from exogenous DNA (11). Their deficiency or malfunction causes autoimmune disorders such as Aicardi-Gutierrez syndrome (12). Increased presence of DNA in sputum from patients with asthma was reported by 2 groups (13, 14). The level of DNA was associated with IL-6 in the sputum and correlated with sputum neutrophils. The relevance of increased sputum DNA for NA remains unknown. In this article, we examine the anti- and pro-inflammatory properties of extracellular DNA and the role of allergens in unmasking its neutrophilic inflammatory activity in asthma.

## Results

### Increased DNA and nucleosomes in body fluids of patients with asthma.

We studied a cohort of patients with NA and non-neutrophilic asthma (N-NA) and a disease control (DC) group. Asthma was defined by the American Thoracic Society (ATS) Expert Panel Report 3 criteria. NA was defined as having a neutrophil proportion of greater than 3% regardless of eosinophil count (*n* ≥ 0) in the bronchoalveolar lavage (BAL). Hence, although referred to as “neutrophilic asthma” for simplicity, people in this study group actually had either neutrophilic or mixed neutrophilic-eosinophilic asthma. Patients with N-NA had less than 3% neutrophils regardless of the eosinophil and lymphocyte counts. This group had eosinophilic and pauci-inflammatory asthma. The DC group included patients with chronic cough, trachea-bronchomalacia, vocal cord dysfunction, and suspected aspiration with negative bronchoscopic findings for aspiration. The demographic characteristics of the patients and DC group are listed in Table 1.

**Figure 1:**
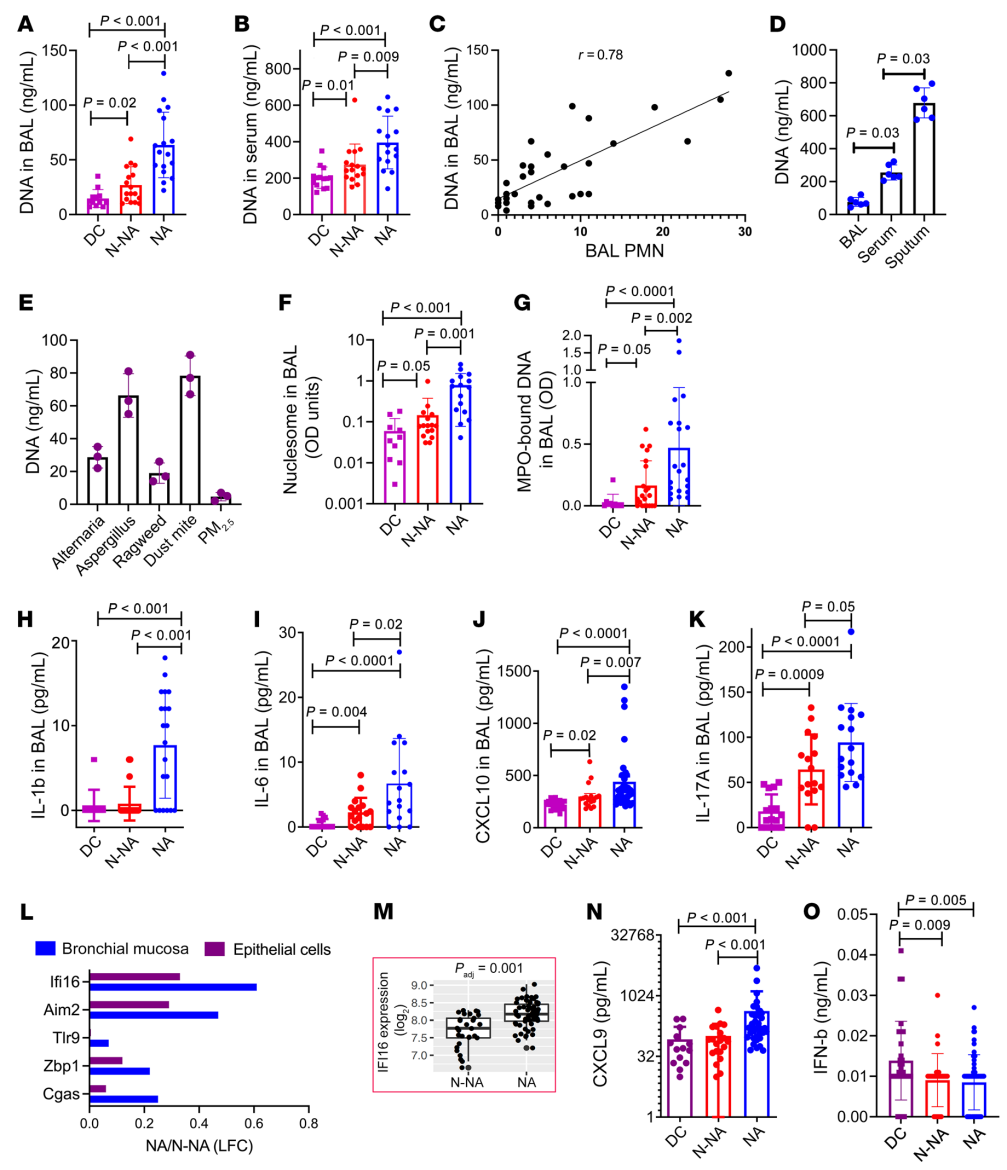
DNA in body fluids and allergens. (**A**) The DNA level in BAL in DC participants (*n* = 12), patients with N-NA (*n* = 17), and those with NA (*n* = 17). (**B**) The DNA level in serum in the DC (*n* = 14), N-NA (*n* = 16), and NA (*n* = 16) groups. (**C**) Correlation analyses between the DNA and PMN in BAL (*n* = 31). Statistical comparisons are between reads from individuals in the DC group and patients with NA. (**D**) Comparison of DNA in BAL, serum, and sputum from patients with asthma (*n* = 6). (**E**) The presence of DNA in allergen extracts and PM_2.5_ (*n* = 3 per group). (**F**) Nuc-bound DNA in BAL from patients in the DC (*n* = 10), N-NA (*n* = 16), and NA (*n* = 16) groups. (**G**) MPO-bound DNA in BAL from patients in the DC (*n* = 10), N-NA (*n* = 21), and NA (*n* = 21) groups. (**H**) The inflammasome cytokine IL-1β in BAL from patients with NA or N-NA, and those in the DC group (*n* = 20 from each type). (**I**) The innate cytokine IL-6 in BAL from patients with NA (*n* = 17) or N-NA (*n* = 17), and those in the DC group (*n* = 17). (**J**) The STING pathway cytokine CXCL10 in BAL from participants in the NA (*n* = 32), N-NA (*n* = 17) and DC (*n* = 18) groups. (**K**) The type 3 cytokine IL-17A in BAL from participants in the NA, N-NA, and DC groups (*n* = 16 with each type). (**L**) Expression of mRNA for DNA sensors in bronchial mucosa and epithelial cells from another cohort of 60 patients with NA versus 30 with N-NA was assessed by microarray, as reported previously (16). The inset (**M**) shows a dot plot of expression of IFI16 mRNA in NA and N-NA. (**N** and **O**) BAL CXCL9 and IFN-β from patients with NA (*n* = 36) or N-NA (*n* = 20) and those with DC (*n* = 14). Data are shown as mean ± SEM. Statistical differences in **A**, **B**, **D**, **E**–**K**, **N**, and **O** were tested using the Mann-Whitney U test. Statistical significance (*P* values) of difference between the groups are shown above the bar graphs. *P*_adj_, adjusted *P* value.

We measured DNA in BAL and serum from this study cohort (Figure 1, A and B). The DNA level was elevated in those with NA as compared with the N-NA and the DC groups. The serum DNA concentration was 10–20 times higher than that in the BAL. Of note, the mucosal lining fluid in BAL is diluted with 120 mL of saline (SAL; the lavage volume). BAL neutrophil counts correlated with the BAL (*r* = 0.78; Figure 1C) but not the serum DNA. BAL DNA did not correlate with serum DNA (*r* = 0.07). We compared the DNA concentration in BAL, serum, and sputum from 6 patients with asthma (Figure 1D). Sputum had the highest level, followed by serum and BAL. This is not a surprise, because sputum is a concentrated form of mucosal lining fluid. In addition, it is likely to contain higher quantities of microbial DNA. BAL DNA correlated with the sputum DNA (*r* = 0.62).

We measured the DNA level in common allergen extracts (namely, *Alternaria*, *Aspergillus*, ragweed, and dust mite) and in the particulate matter of 2.5 μm (PM_2.5_; from the US National Institute of Standards and Technology). The allergen extracts contained low levels of DNA (Figure 1E). Stretches of mammalian DNA wrap around a bundle of 8 histones and are known as nucleosomes (Nucs). We measured Nuc in BAL, using a modified ELISA that simultaneously detects DNA and histones (Cell Death Detection ELISA Plus; Roche). BAL contained low levels of Nuc, which were significantly elevated in NA (Figure 1F) and marginally elevated in N-NA.

Extracellular DNA originates from cells dying as a result of physiological turnover (8). Neutrophils release DNA that is bound to myeloperoxidase (MPO) during generation of neutrophil extracellular trap (NET). We detected significantly higher quantities of MPO-bound DNA in BAL from the NA group as compared with the N-NA and DC groups (Figure 1G). Internalized DNA activates inflammasomes and the stimulator of interferon genes (STING) and the innate immune pathways (15). We measured the inflammasome product IL-1β, the STING-regulated CXCL10, and the innate/type-3 cytokines IL-6 and IL-17A. They were increased significantly in BAL from patients with NA and correlated with BAL PMNs (Figure 1, H–K). The correlation coefficients (*r* values) were 0.73 for IL-1β, 0.51 for CXCL10, 0.66 for IL-6, and 0.62 for IL-17A.

### Higher concentration of DNA sensors and their regulated cytokines in the airway tissue of patients with asthma.

Internalized DNA/Nuc is destroyed by DNases (11). Any leftover DNA is detected by DNA sensors, that is, the inflammasome DNA sensors IFI16 and Aim2, and the noninflammasome DNA sensors cGAS, TLR9, ZBP1, among others (15).

We previously studied a cohort of 60 patients with refractory asthma, 30 patients with well-controlled asthma, and 20 healthy control participants (4). Refractory asthma was associated with increased airway neutrophils. As a part of this project (4), we examined the transcriptome of airway epithelial cells (bronchial brushing) and bronchial mucosa (endobronchial biopsy) by microarray (Affymetrix Hu133A 2.0) (16). The results showed modest but significantly increased expression of IFI16 in both bronchial mucosa and epithelial cells from NA (Figure 1L and Figure 1M). AIM2 and cGAS were elevated in epithelial cells only. IFI16, cGAS, and ZBP1 signal through STING, which induces a cytokine profile that includes CXCL9 and IFN-β (9, 15). CXCL9 was increased and IFN-β was decreased in NA (Figure 1, N and O) suggesting that the antiinflammatory function of STING was selectively suppressed.

### Inhaled DNA and Nuc are noninflammatory, but their latent neutrophilic inflammatory capacity is unmasked by the allergen Alternaria.

**Figure 2:**
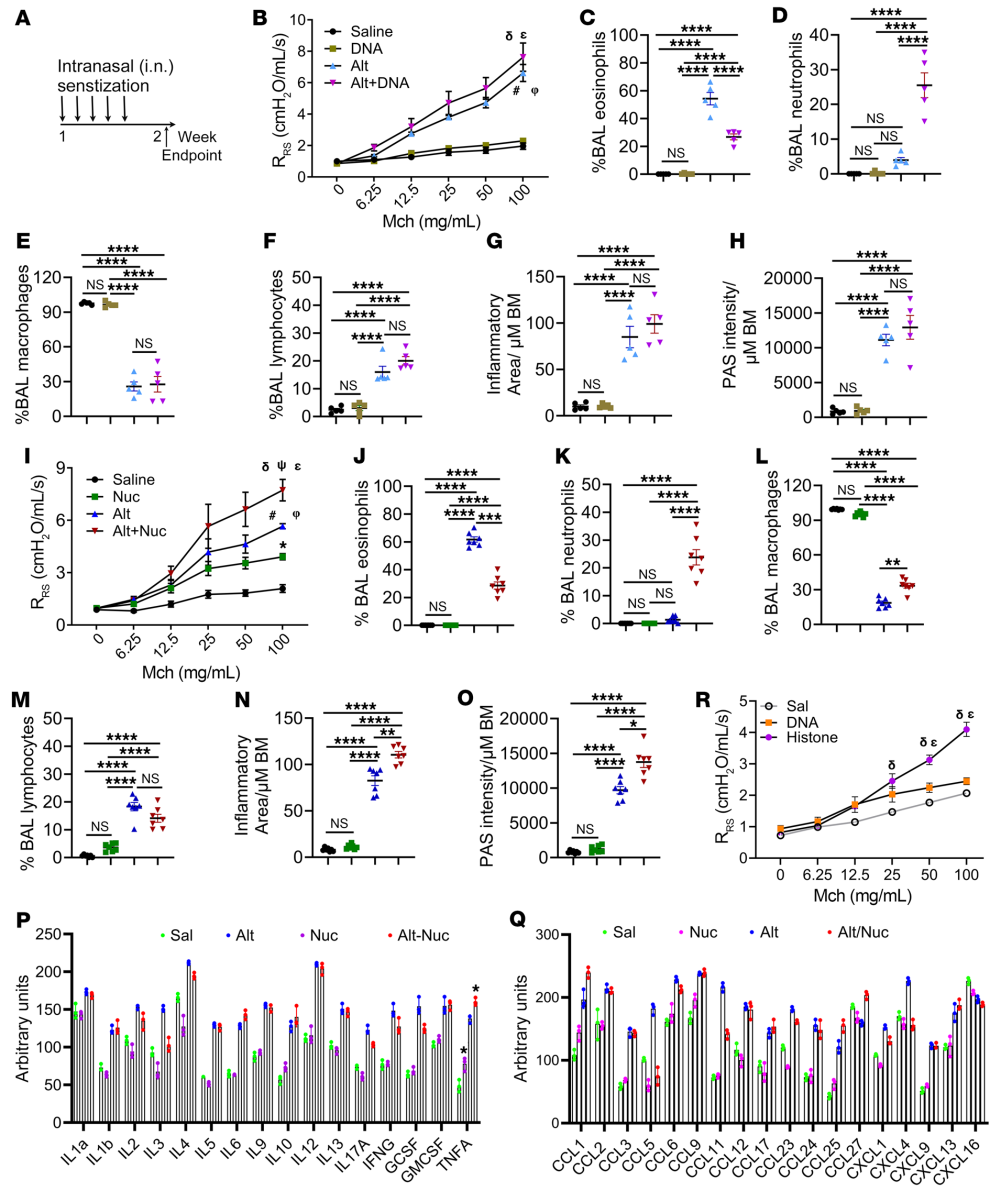
Effect of i.n. exposure to DNA, Nuc, and Alt (alone or in combination) on mouse AHR, inflammation, and mucus production. (**A**) A schematic of the i.n. administration of various agents and the experimental end point. (**B**) AHR after Sal, DNA, Alt, or Alt+DNA exposure as measured by flexiVent. ^ε^Sal versus Alt+DNA, *P* < 0.0001; ^φ^Sal versus Alt, *P* < 0.0001; ^#^DNA versus Alt, *P =* 0.0001; ^δ^DNA versus Alt+DNA, *P* < 0.0001. (**C**–**F**) Differential counts of BAL macrophages, lymphocytes, eosinophils, and neutrophils (*n* = 5). (**G** and **H**) Morphometric quantification of peribronchial and perivascular inflammation (H&E staining of the lungs) and mucus production (PAS staining) in Sal-, DNA-, Alt-, or Alt+DNA–treated mice (*n* = 5). (Groups are color-coded as in **B**). (**I**) AHR after Sal, Nuc, Alt, or Alt-Nuc exposure as measured by flexiVent. R_RS_, respiratory system resistance. *Sal versus Nuc, *P* = 0.02; ^φ^Sal versus Alt, *P* < 0.0001; ^ε^Sal versus Alt-Nuc, *P* < 0.0001; ^#^Nuc versus Alt, *P =* 0.02; ^δ^Nuc versus Alt-Nuc, *P* < 0.0001; ^ψ^Alt versus Alt-Nuc, *P* = 0.006. (*n* = 7). (**J**–**M**) Differential counts of BAL macrophages, lymphocytes, eosinophils, and neutrophils (*n* = 7). (**N** and **O**) Morphometric quantification of peribronchial and perivascular inflammation and mucus production in mice treated with Sal, Nuc, Alt, or Alt-Nuc (*n* = 7). BM, basement membrane. (Groups are color coded as in **I**). (**P** and **Q**) Expression (semi-quantitative) of select cytokines and chemokines in BAL from mice treated with Sal, Nuc, Alt or Alt-Nuc, as measured by a cytokine array (*n* = 3). (**R**) AHR after Sal, DNA, or histone exposure in B6 mice (*n* = 5). ^δ^Sal versus histone, *P* < 0.001; ^ε^DNA versus histone, *P* < 0.001. A 2-way ANOVA with Tukey’s multiple comparisons test was used to determine the statistical significance between groups. Data are presented as mean ± SEM. *P < 0.05, **P < 0.01, ***P < 0.001. ****P < 0.0001.

We administered Sal i.n., the *Alternaria* allergen (Alt), DNA, or Alt and DNA in combination (Alt-DNA) for 5 consecutive days to 4 groups of female B6 mice, as shown in Figure 2A. This short 5-day protocol was designed to elicit primarily an innate immune response. We measured airway hyperreactivity (AHR) and other features of asthma (i.e., inflammation, mucus) 2 days later (Figure 2, B–H). Alt and Alt-DNA, but not DNA, induced AHR as compared with Sal (Figure 2B). Alt induced eosinophilic influx (Figure 2C). DNA alone did not induce inflammation. However, when coadministered with Alt, DNA induced neutrophilic influx and reduced Alt-induced eosinophils in BAL. Alt and Alt-DNA induced similar levels of peribronchial/perivascular inflammation and mucus production (Figure 2, G and H, and Supplemental Figure 1A; supplemental material available online with this article; https://doi.org/10.1172/JCI187907DS1).

Next, we examined the effect of Nuc, using the same protocol. Unlike DNA, Nuc alone induced modest AHR, like DNA, however, Nuc did not induce granulocytic inflammation or mucus production (Figure 2, I–O, and Supplemental Figure 1B). Nuc increased lymphocyte influx into the airway lumen (increase in BAL). However, it did not increase peribronchial or perivascular inflammation. Simultaneous administration of Alt and Nuc (Alt-Nuc) resulted in higher AHR, mixed neutrophilic-eosinophilic inflammation, and increased mucus production as compared with Alt. To determine if Nuc could affect the asthma phenotype for a different allergen, we used the allergen extract of *Aspergillus fumigatus* (Asp). Nuc alone induced mild AHR but no inflammation (Supplemental Figure 1, C–I). As expected, Asp induced AHR and eosinophilic, but not neutrophilic, inflammation. The Asp-Nuc combination induced AHR, and mixed eosinophilic-neutrophilic inflammation. The results suggest that the ability of Nuc to induce neutrophilic inflammation is not restricted to Alt.

### Nuc induced innate cytokines but inhibited type 1, 2, and 3 cytokines.

We semi-quantitatively examined cytokines and chemokines in the BAL fluid (BALF) from the mouse model by an array (Figure 2, P and Q). Nuc alone induced CCL1, CCL9, and TNF-α but inhibited IL-4, CCL5, CCL20, CCL23, CXCL1, CXCL5, and VCAM1. Alt robustly induced nearly all cytokines and chemokines except CCL27 and CXCL16. Alt-Nuc further augmented the production of CCL1, CCL27, CXCL9, IL-1β, IL-6, and TNF-α but inhibited CCL5, CCL11, CXCL4, IL-3, IL-4, IL-17A, IFN-γ, and GCSF when compared with Alt alone. The results suggested that Nuc inhibited Alt-induced type 1 (CCL5, IFN-γ), type 2 (CCL11 and IL-4), and type 3 (IL-17A) responses, whereas it amplified the innate (IL-1β, IL-6, and TNF-α) response. Nucs roughly contain an equal mass of histones and DNA. We administered 5 μg of histones and 5 μg of DNA to separate groups of mice (see Figure 2A) and compared their AHR. Histones, but not DNA, induced significant AHR (Figure 2R).

### Nuc alone induced antiinflammatory and defensive genes but stimulated inflammatory (pro-neutrophilic) genes when coadministered with Alt.

**Figure 3:**
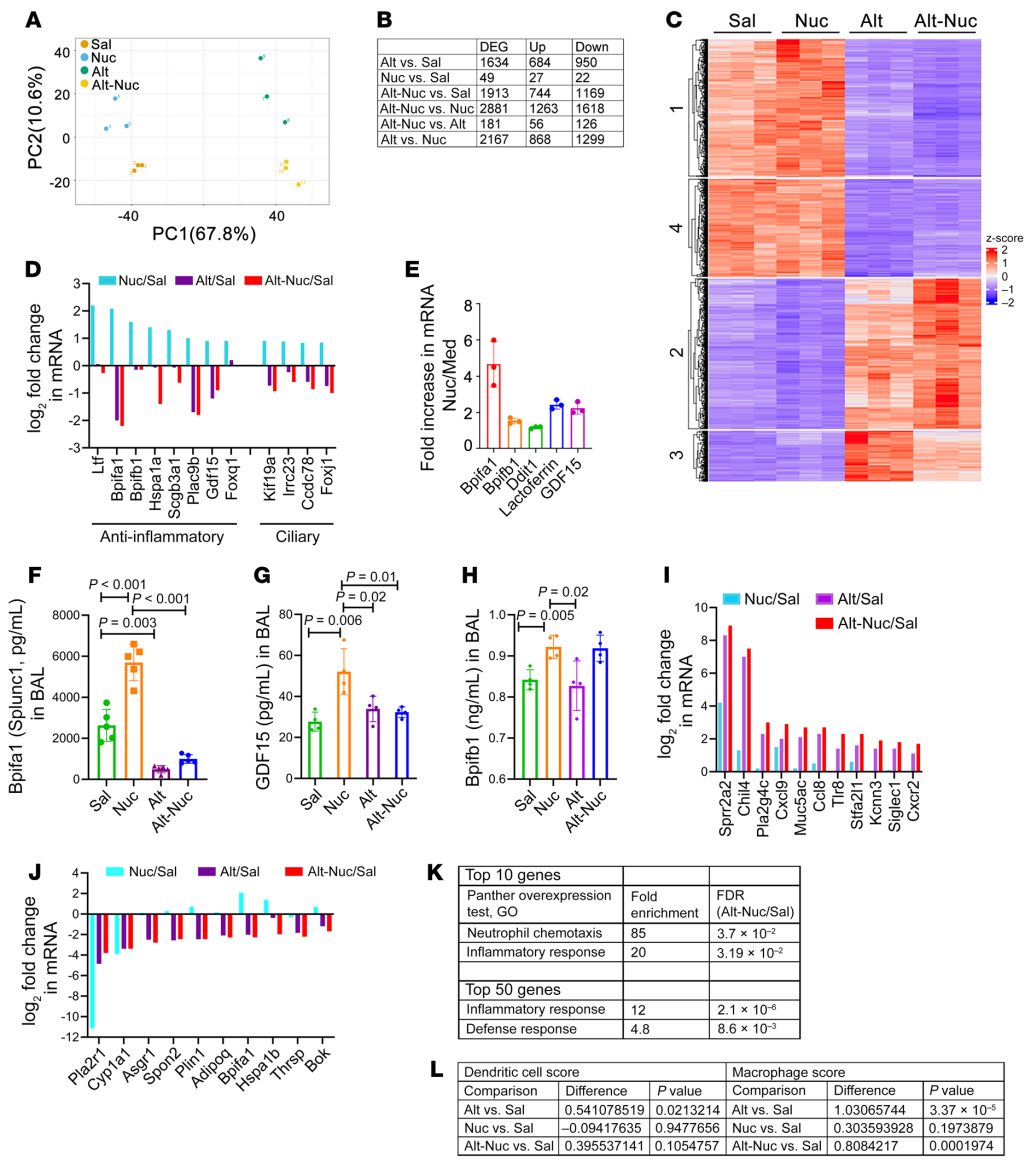
Transcriptomic analysis of the mouse models. (**A**) Principal component (PC) analysis of transcriptomic changes (RNA-Seq of the lung tissue) in Sal-, Nuc-, Alt-, and Alt-Nuc–treated mice (*n* = 3). (**B**) Comparison of differentially expressed genes and up- and downregulated genes between the study groups. (**C**) Heat map of top 200 genes among the 4 study groups. (**D**) Top genes selectively induced by Nuc and inhibited by Alt and Alt-Nuc. (**E**) Validation of increased mRNA (RT-PCR) expression of *Bpifa1*, *Bpifb1*, *Ddit1*, Lactoferrin, and *GDF15* in Nuc- versus medium-treated mouse airway epithelial cells. Med, medium. (**F**–**H**) Measurement of BPIFA1, GDF15, and BPIFB1 in BAL by ELISA from Sal-, Nuc-, Alt- and Alt-Nuc–treated mice (*n* = 4–5). (**I** and **J**) Top upregulated (**I**) and downregulated (**J**) genes (Log2 fold change compared with Sal) among the study groups. (**K**) Top biological processes driven by the top 50 and top 10 genes from the study groups. GO, Gene Ontology. (**L**) Comparison of dendritic cell versus macrophage scores for association with the transcriptomes from the study groups. A 2-way ANOVA with Tukey’s multiple comparisons test were used to determine the statistical significance between groups. Data are presented as mean ± SEM. Statistical significance (*P* values) of difference between the groups are shown above the bar graphs.

We performed RNA-Seq of the lung tissue from the mouse models. Principal component analysis showed that the transcriptomic profile of the lung tissue from Sal, Nuc, Alt, and Alt-Nuc groups differed (Figure 3A). The Nuc group clustered close to the Sal group, and the Alt group clustered close to the Alt-Nuc group. The differential gene expression (DEG) among the study groups is shown in Figure 3B. The largest difference in DEG was seen in the Alt-Nuc group, followed by the Alt group. The Nuc group had the lowest DEG. A heat map of top DEGs from the 4 study groups is shown in Figure 3C.

The top DEGs in the Nuc versus Sal groups are shown in Figure 3D. Nuc upregulated epithelial antiinflammatory/defensive genes *Ltf, Bpifa1, Bpifb1, Hspa1a, Scgb3a1, Plac9a, Gdf15*, and *Foxq1* (17, 18) and ciliary (defensive) genes *Kif19a, Lrrc23, Ccdc78*, and *Foxj1* (19, 20). Lactoferrin (LTF), SCGB3A1 (high in normal-1), and especially, BPIFA1 (short palate lung and nasal epithelial clone 1), and BPIFB1 (long-PLUNC1), are potent antimicrobial and antiinflammatory proteins (17, 18). The Nuc-induced cilia genes also promote antibacterial defense and prevent inflammation. Strikingly, Alt inhibited the expression of the antiinflammatory genes (Figure 3D). Furthermore, Alt overrode the antiinflammatory gene–inducing effect of Nuc. We confirmed increased expression of select genes in Nuc-treated primary bronchial epithelial cells (Figure 3E). We also confirmed increased levels of the BPIFA1, GDF15, and the BPIFB1 in BAL from the Nuc model (Figure 3, F–H). Alt inhibited Nuc-stimulated production of the antiinflammatory molecules BPIFA1 and GDF15 but not BPIFB1, which was in agreement with the RNA-Seq data (Figure 3D). Nuc upregulated smooth muscle–related genes *Fbxl22*, *Myh11*, *Nkx2-5*, *Mylk2*, and *Actg2* (Supplemental Figure 2A) (21, 22). It also upregulated *H19*, *Neb*, *Tnnt3*, and *Casq1*, but this was not specific, because these genes were also increased by Alt (Supplemental Figure 2B). Increased expression of these genes could promote smooth muscle hypertrophy and explain AHR induction by Nuc. The Nuc-induced downregulated genes are shown in Supplemental Figure 2C.

Because the addition of Nuc superimposed neutrophilic inflammation on Alt-induced eosinophilic inflammation, we examined the top DEGs that were induced or inhibited in the Alt-Nuc group as compared with the Alt and Nuc groups. The top upregulated genes in the Alt-Nuc group included *Sprr2a2*, *Chil4*, *Pla2g4c*, *Cxcl9*, *Tlr8*, *Stfa2l1*, *Kcnn3*, *Siglec1*, and *Cxcr2* (Figure 3I). The foregoing genes are broadly involved in macrophage activation and neutrophilic inflammation (23–25). Their higher expression pointed to macrophage-driven neutrophilic inflammation, which was validated by our histologic and BAL cell data (Figure 2). The top downregulated genes were *Pla2r1*, *Cyp1a1*, *Asgr1, Spon2, Plin2, Adipoq, Bpifa1, Hspa1b, Thrsp*, and *Bok* (Figure 3J). The foregoing genes are involved in inhibition of inflammation, apoptosis, and lipid metabolism (26–28). Inhibition of the foregoing antiinflammatory genes would likely promote inflammation. A Panther test of the top DEGs from Alt-Nuc versus Sal showed an overrepresentation of biological processes related to inflammatory and defense responses, innate immune cells, and neutrophil/macrophage chemotaxis (Figure 3K). Overrepresented processes in the DEG from Alt- Nuc versus Alt were actin cytoskeleton, innate immune response and NF-kB signaling (Supplemental Figure 2D). A cell identification score analysis of the RNA-Seq showed that macrophages had high scores for Alt and Alt-Nuc (Figure 3L). Dendritic cells showed statistical significance for Alt only. The foregoing results suggested that airway macrophages primarily responded to the Alt-Nuc challenge.

### Inhaled Nucs were internalized by macrophages and innate lymphoid cells.

**Figure 4:**
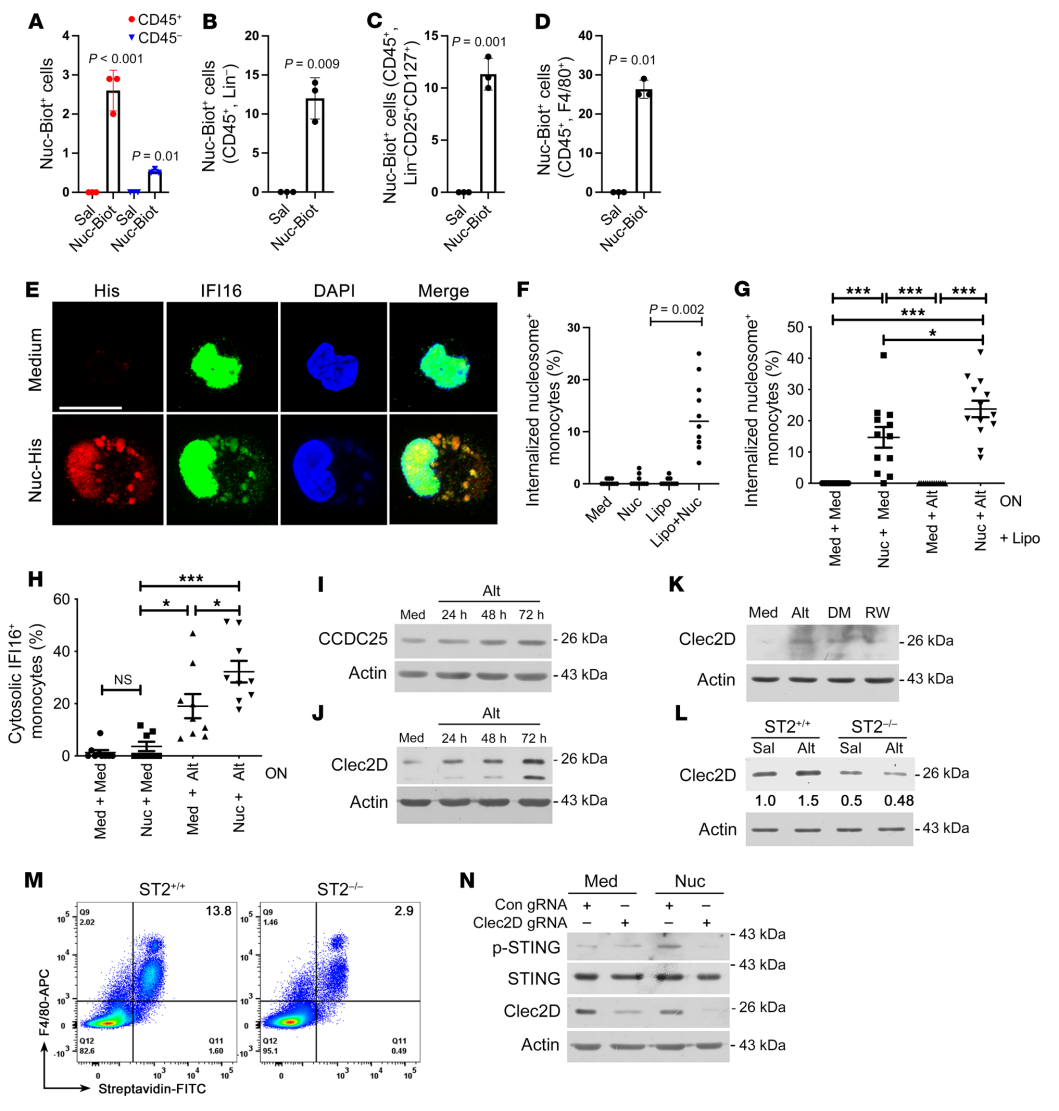
Nuc uptake. (**A**–**D**) Labeled Nuc (Nuc-biotin/His; 10 μg/mouse) or Sal were administered i.n. to mice (*n* = 3), and biotin^+^ cells were analyzed by FCM in various lung cell populations. (**E**) Healthy cadaveric alveolar macrophages were incubated with His-labeled Nuc for 4 hours and immunostained for His (red, cytosolic DNA), IFI16 (green), and nucleus (blue). Scale bar, 5 μm (**F**) Isolated blood monocytes from healthy donors (*n* = 10) were incubated with His/biotin-labeled Nuc (with or without lipofectamine [Lipo]) for 4 hours and cells with internalized Nuc (His^+^) were quantified. (**G**) Blood monocytes from patients with asthma (*n* = 12) were cultured overnight (ON) with medium (Med) or Alt (5 μg/mL) and then incubated with His-labeled Nuc in the presence of Lipo for 4 hour. Cytosolic Nuc^+^ (His^+^) cells were analyzed. (**H**) Blood monocytes from patients with asthma (*n* = 9) were cultured as above but without Lipo. Cells were immunostained for IFI16^+^. Cytosolic IFI16^+^ monocytes were analyzed. (**I**–**K**) Healthy cadaveric alveolar macrophages were incubated with Alt for an increasing time (**I** and **J**), or with Alt, dust mites (DM), and ragweed (RW) (**K**) for 72 hours and then immunoblotted for CCDC25 (**I**) or CLEC2D (**J** and **K**) (*n* = 3). (**L**) BMDM from WT and ST2*^–/–^* mice were cultured with Sal or Alt (5 μg/mL) for 48 hours and then immunoblotted for CLEC2D (*n* = 3). (**M**) Internalization of Nuc-biotin by WT and ST2*^–/–^* BMDMs was detected by FCM (*n* = 3). (**N**) Cadaveric alveolar macrophages were transfected with control (Con) gRNA or CLEC2D gRNA. Cells were incubated 48 hours later with medium or Nuc for 4 hours and immunoblotted for CLEC2D and p-STING (*n* = 3). Comparison made by Student’s 2-tailed *t* test (**A**–**D**) and 2-way ANOVA with Tukey’s multiple comparisons test (**F**–**H**). Data are presented as mean ± SEM. *P < 0.05, ***P < 0.001.

We examined the lung cells that internalized i.n. administered Nuc. We administered His-tagged and biotinylated recombinant Nuc to mice and examined lung cells by flow cytometry (FCM) 4 hours later. We detected 2.5% and 0.5% of CD45^+^ and CD45^–^ lung cells, respectively, positive for Nuc (Figure 4, A and B). Of the CD45^+^ cells, 26% of F4/80 macrophages and 12% of Lin-ILCs were positive for Nuc (Figure 4, C and D). We examined activation of airway macrophages by Nuc. We cultured BAL macrophages from a patient in the DC group with medium or Nuc for 4 and 24 hours. The secretion of 80 cytokines and chemokines was measured by the human cytokine array C5 (RayBiotec, Inc.) Nuc increased the production of TNF-α, IL-6, IL-10, CCL7, CCL8, CXCL9, and CXCL10 (Supplemental Figure 3).

### Alt facilitated Nuc/DNA internalization by blood monocytes and airway macrophages.

We examined His-tagged Nuc uptake in vitro by PBMCs and BAL macrophages from patients with asthma and alveolar macrophages from cadaveric lungs obtained from healthy individuals. The internalized DNA was detected by immunostaining of cytosolic His-tag, the presence of extranuclear DAPI staining, and by immunostaining with an Ab that specifically detects non-nuclear dsDNA. Alveolar macrophages internalized Nuc within 4 hours (Figure 4E), as detected by the His tag and cytosolic DAPI staining. IFI16 is physiologically localized to the nucleus.

The presence of cytosolic DNA triggers translocation of IFI16 to the cytosol. We detected cytosolic translocation of IFI16 and colocalization with Nuc-His and cytosolic DAPI. The internalized Nuc was also detected by staining with an anti-dsDNA Ab (Supplemental Figure 4A). Unlike lung macrophages, plastic-adherent blood monocytes did not internalize Nuc but did so in presence of lipofectamine (Figure 4F). This facilitated internalization was significantly increased by Alt (Figure 4G). An overnight preincubation with Alt was sufficient for Nuc internalization and IFI16 cytosolic translocation in monocytes in the absence of lipofectamine (Figure 4H). We examined the functional relevance of Alt-Nuc co-operation by measuring IL-1β, IL-6, and TNF-α. Nuc alone did not induce these cytokines. Alt induced all 3 cytokines. The Alt-Nuc combination had higher production IL-1β and TNF-α, but not IL-6, as compared with Alt (Supplemental Figure 4, B–D).

### Alt upregulated the DNA/Nuc receptors CCDC25 and CLEC2D and facilitated Nuc activation of STING.

DNA is internalized by the receptor CCDC25 (29), and Nucs are internalized by CLEC2D (30). Because Alt facilitated Nuc uptake and its biological outcomes, we examined the effect of Alt on CCDC25 and CLEC2D expression by BAL macrophages from healthy human cadaveric lungs. Alt increased macrophage expression of CCDC25, and, especially, CLEC2D (Figure 4, I and J, and Supplemental Figure 5). Like Alt, dust mite and ragweed allergens also increased CLEC2D (Figure 4K). Allergens induced IL-33 (Supplemental Figure 4E), which activates interleukin 1 receptor–like 1 (also known as IL1RL1 and ST2) (31). We asked if ST2 was involved in CLEC2D upregulation. Bone marrow–derived macrophages (BMDM) from ST2*^–/–^* mice had reduced expression of CLEC2D at baseline (Figure 4L). Alt upregulated CLEC2D in WT but not in ST2*^–/–^* BMDM. This reduced CLEC2D expression severely impaired Nuc-biotin uptake by F4/80^+^ lung macrophages (Figure 4M).

The detection of cytosolic DNA by DNA sensors activates and phosphorylates STING. We examined colocalization of phospho-STING (p-STING), IFI16, and CLEC2D in Nuc-treated cadaveric BAL macrophages by triple immunostaining. Internalized Nuc colocalized with endocytosed CLEC2D, cytosolic p-STING, and extranuclear IFI16 in Nuc-treated macrophages (Supplemental Figure 6A). We examined the importance of CLEC2D for Nuc internalization and STING phosphorylation by gene deletion. Guide RNA (gRNA)-mediated depletion of CLEC2D largely abolished Nuc-induced activation of STING (p-STING) in macrophages (Figure 4N), underscoring the importance of CLEC2D.

### Cytosolic IFI16 and p-STING were increased in airway cells from patients with NA.

**Figure 5:**
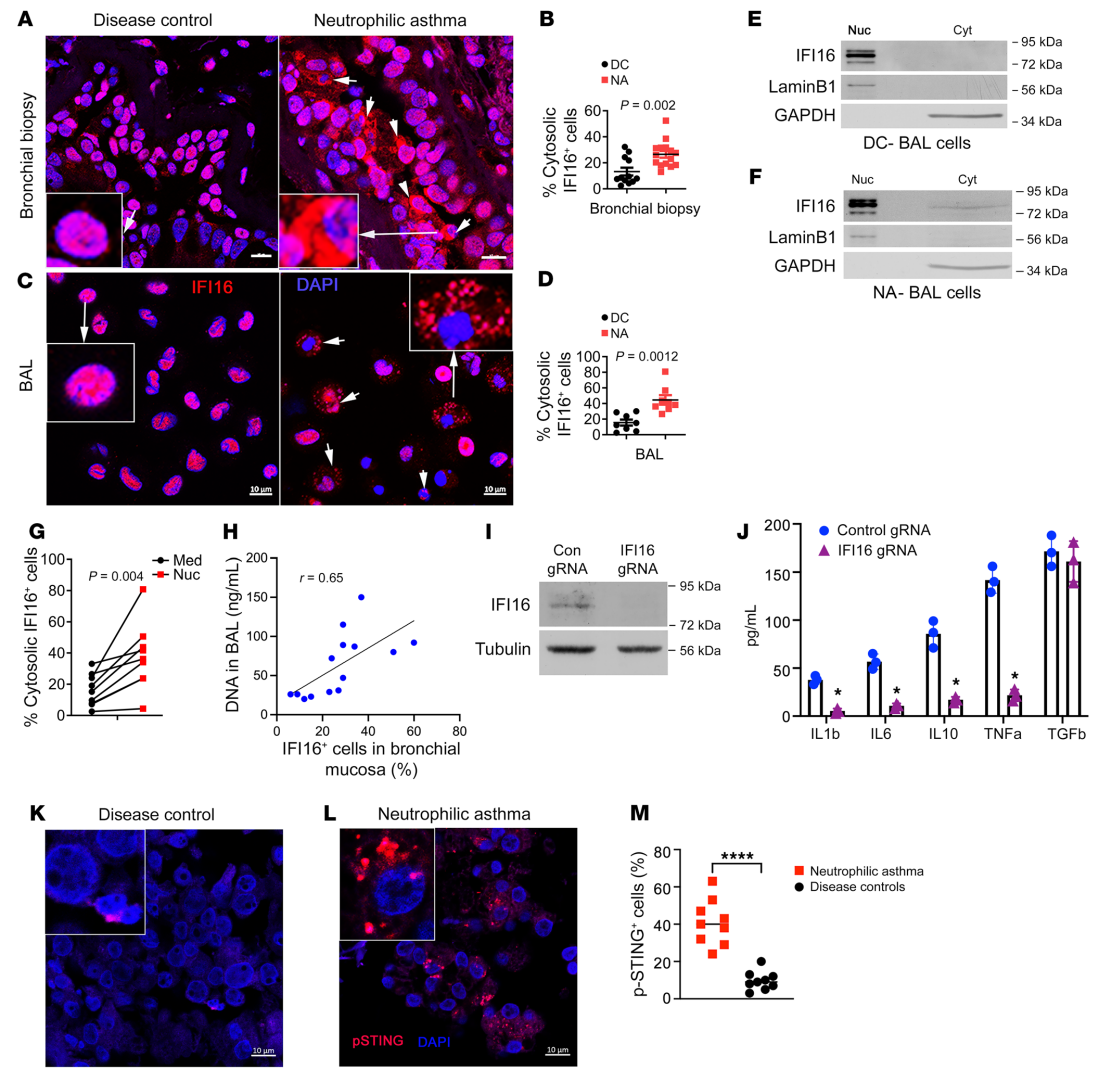
Cytosolic IFI16 and activated STING in NA. (**A**–**D**) Representative image of endobronchial biopsy (*n* = 12 per group) (**A**) and BAL samples (*n* = 8 per group) (**C**) from patients with NA and those in the DC group. Specimens were immunostained for IFI16 (red) and counterstained with DAPI (blue). Extranuclear IFI16^+^ (arrows) cells were compared and quantified between the 2 study groups (**B** and **D**). Comparison was made by Student’s 2-tailed *t* test. Scale bar, 10 μm. (**E** and **F**) BAL cells from a participants in the DC group and from a patient with NA were fractionated for nuclear and cytosolic fractions and immunoblotted for IFI16. Lamin B and GAPDH were used as markers for nuclear and cytosolic fractions, respectively. Asthmatic BAL cells showed cytosolic IFI16 (*n* = 3). (**G**) BAL cells from select patients with asthma were incubated with Nuc in vitro for 4 hours and then stained for IFI16. IFI16^+^ cells were quantified and statistically analyzed. Comparison was made by paired *t* test. (**H**) Correlation of IFI16^+^ cells in the bronchial mucosa (**A** and **B**) with DNA levels in the corresponding BAL. (**I** and **J**) Monocyte-derived macrophages (MDM) were transduced with control (Con) or IFI16 gRNA along with CRISPR-Cas9 and then immunoblotted for IFI16 and reprobed for tubulin. (**J**) MDMs transduced with control or IFI16 gRNA were cultured for 24 hours, and culture supernatant was assayed for the indicated cytokines (*n* = 3). Comparison was made by Student’s 2-tailed *t* test. *P < 0.05. (**K**–**M**) Endobronchial biopsy samples from the DC participants and patients with NA were stained for p-STING, and the quantified data compared between the study groups. ****P < 0.0001 by Student’s 2-tailed *t* test. Scale bar, 10 μm. Med, medium.

Immunostaining showed decreased nuclear and correspondingly increased cytosolic IFI16 (white arrows) in the airway tissue (from bronchial biopsy specimens) and BAL macrophages from patients with NA as compared with the DC group (Figure 5, A–D). A careful examination of the biopsy samples showed extranuclear DAPI-positive structures that colocalized with extranuclear IFI16 (Supplemental Figure 7A). The presence and absence of cytosolic IFI16 in NA and DC BAL cells were confirmed by subcellular fractionation and immunoblotting (Figure 5, E and F).

We cultured BAL macrophages from NA with Nuc overnight and examined cytosolic IFI16^+^ cells. BAL macrophages from NA had baseline (medium cultured) elevated cytosolic IFI16^+^ cells, which were further augmented upon culture with Nuc (Figure 5G). We observed a positive correlation (*r* = 0.65) between BAL DNA and cytosolic IFI16^+^ cells in biopsy samples (Figure 5H). We stained cadaveric lung tissue from 4 healthy individuals and 4 patients who had fatal asthma for IFI16 and p-STING. IFI16 was localized to the nucleus in cells from healthy cadaveric lungs (Supplemental Figure 7B). In contrast, IFI16 was mostly extranuclear and cytosolic in fatal asthma. The overall expression of IFI16 was increased in fatal asthma. p-STING expression was also increased in the lung specimens from patients with fatal asthma (Supplemental Figure 7C).

We examined the relevance of IFI16 activation for macrophage cytokine production. Monocyte-derived macrophages were transfected with a gRNA targeting IFI16, a control gRNA, and CRISPR-Cas9 plasmid, and examined for IFI16 gene deletion by immunoblotting and cytokine production by ELISA. IFI16 gRNA-mediated gene deletion resulted in loss of protein expression (Figure 5I), which was associated with inhibition of production of IL-1β, IL-6, IL-10, and TNF-α but not TGF-β (Figure 5J). We immunostained bronchial biopsy samples for p-STING. Patients with NA had increased frequency of p-STING^+^ cells in the bronchial mucosa as compared with the DC group (Figure 5, K–M). There was a strong correlation between p-STING^+^ cells and BAL DNA (*r* = 0.72; *n* = 9).

### STING and its downstream effectors were activated in Alt-Nuc–treated lung cells.

**Figure 6:**
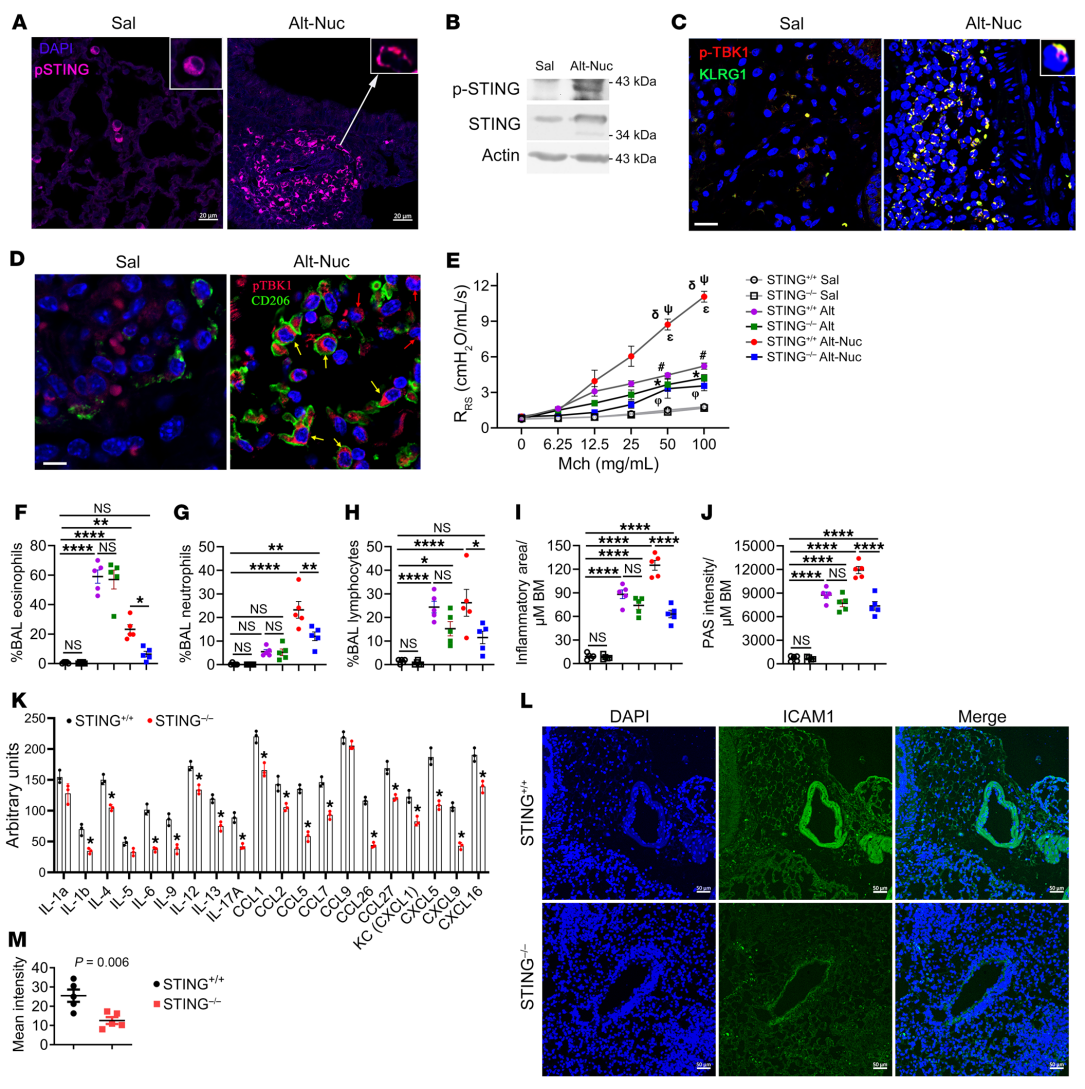
Role of STING in the Alt-Nuc and Alt models of asthma. (**A**) Expression of p-STING in the lung. Mouse lungs from the Sal and Alt-Nuc models were immunostained for p-STING (red) and counterstained for DAPI (*n* = 3). Scale bar 20 μm. (**B**) Immunoblot analysis of the lung tissue from the Sal and Alt-Nuc models for p-STING (*n* = 3). The membranes were reprobed for STING and actin. (**C**) Lung specimens were immunostained for p-TBK1 (red) and KLRG1 (a marker of ILC2s, and a fraction of activated NK cells and T cells) (green). Scale bar, 10 μm. (**D**) Lung specimens were double stained for p-TBK1 (red) and CD206 (a macrophage marker) (green) (*n* = 3). Scale bar, 5 μm. (**E**) AHR after Alt-Nuc treatment (see Figure 2A) of *Sting*^+/+^ and *Sting^–/–^* mice as measured by flexiVent. ^ε^*Sting*^+/+^-Sal versus *Sting*^+/+^-Alt-Nuc, *P* < 0.0001; *Sting^+/+^-Sal versus *Sting*^+/+^-Alt, *P* < 0.0001; ^φ^*Sting*^+/+^-Sal versus *Sting^–/–^*-Alt, *P* = 0.002; ^δ^*Sting*^+/+^-Alt versus *Sting*^+/+^-Alt-Nuc, *P* <0.0001; ^Ψ^*Sting^–/–^*-Alt-Nuc versus *Sting*^+/+^-Alt-Nuc, *P* < 0.0001; ^#^*Sting^–/–^*-Sal versus *Sting^–/–^*-Alt, *P* = 0.001. (*n* = 6; 2-way ANOVA with Tukey’s multiple comparisons test). (**F**–**H**) Differential counts of BAL lymphocytes, eosinophils, and neutrophils. (**I** and **J**) Morphometric quantification of inflammation and mucus production in the lung tissue from *Sting*^+/+^ and *Sting^–/–^* mice from the study groups. (*n* = 5; 2-way ANOVA with Tukey’s multiple comparisons test). BM, basement membrane. (**K**) Expression (semi-quantitative) of select cytokines and chemokines in BAL from *Sting*^+/+^ and *Sting^–/–^* mice treated with Alt-Nuc as measured by a cytokine array. Comparison made by Student’s 2-tailed *t* test. *P < 0.05. (**L** and **M**) ICAM1 immunostaining (green) of the lung tissue and the quantification of MFI of the stained blood vessels from *Sting*^+/+^ and *Sting^–/–^* mice (*n* = 5). Scale bar, 50 μm. Comparison made by Student’s 2-tailed *t* test. Data are presented as mean ± SEM. *P < 0.05, **P < 0.01, ****P < 0.0001.

Immunostaining of the lung tissue showed significant p-STING staining in peribronchial inflammatory cells (Figure 6A) in the lung from the Alt-Nuc– but not Sal-treated mice. We confirmed the increased p-STING expression in the Alt-Nuc lung by immunoblotting (Figure 6B). The activation of STING leads to activation and phosphorylation of TBK1 (9). We double immunostained the lung tissue for p-TBK1 and the ILC marker KLRG1 or the macrophage marker CD206. The majority of p-TBK1^+^ cells costained with KLRG1 (Figure 6C). Note that KLRG1 also stains highly activated CD8 T cells and NK cells. We detected p-TBK1 in CD206^+^ and CD206^–^ macrophages (Figure 6D). Noncanonical autophagy is a conserved function of STING (32). We examined autophagy by immunostaining of the lung tissue for LC3B. Sal- and Nuc-treated mice did not have autophagy (Supplemental Figure 8). Alt alone induced autophagy in airway cells. The addition of Nuc to Alt significantly increased autophagy, indicating a positive functional interaction.

### STING and upstream DNA sensors were essential for Alt-Nuc–induced neutrophilic- eosinophilic asthma.

To demonstrate the role of STING, we studied a germline gene–deleted mouse strain (33). *Sting^–/–^* mice exposed to Alt-Nuc showed inhibition of AHR, eosinophilic, neutrophilic, and lymphocytic infiltrates in BAL; airway inflammation; and mucus production as compared with *Sting^+/+^*mice (Figure 6, E–J). The foregoing was in contrast with the results from the Alt-alone asthma model. *Sting^–/–^* mice had no significant change in AHR, inflammation, or mucus production in the Alt model. *Sting^+/+^* and *Sting^–/–^* mice that were exposed to Sal in control experiments did not have AHR or inflammatory changes.

To establish the translational relevance of STING, we pretreated mice with the STING inhibitor H151 (750 nmol per mouse; ref. 34) 1 hour before Alt-Nuc treatment (Figure 2A). The STING inhibitor attenuated AHR and neutrophilic, but not eosinophilic, inflammation (Supplemental Figure 9, A–C). A cytokine array of the BAL from the Alt-Nuc model showed inhibition of cytokines and chemokines belonging to innate, types 1, 2, and 3 immune responses (Figure 6K) in *Sting^–/–^* mice. The inhibition of inflammation suggested vascular impermeability. We stained the lung tissue for ICAM1. The lung blood vessels from Alt-Nuc–treated WT mice showed robust staining for ICAM1, which was negligibly present in *Sting^–/–^* mice (Figure 6, L and M).

**Figure 7:**
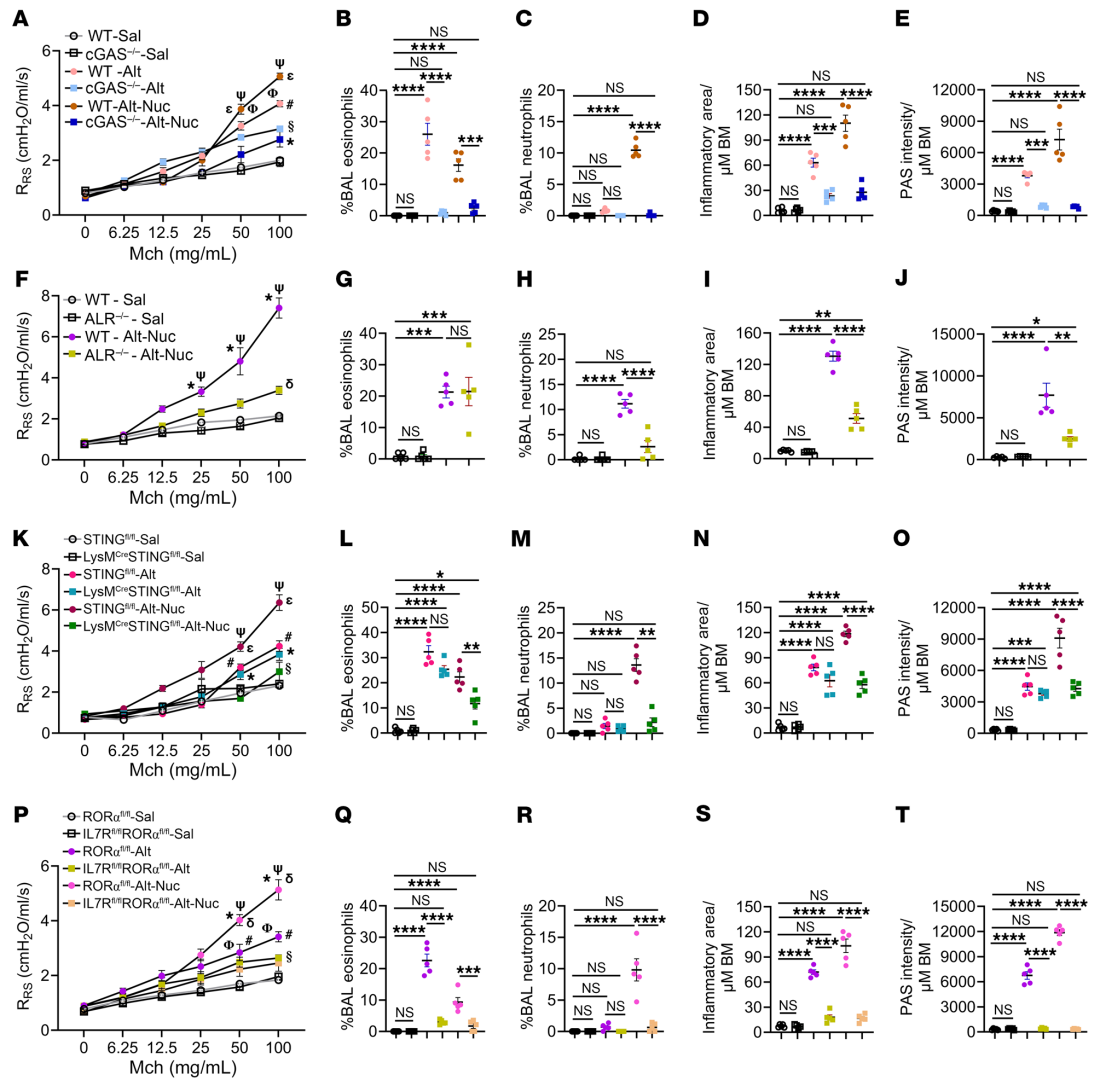
Effect of germline deletion of various DNA sensors and ILC2s on Alt-Nuc and Alt-induced asthma. (**A**) AHR after Alt-Nuc, Alt, and Sal exposure in WT and *Cgas^–/–^* mice (*n* = 5) as measured by flexiVent. R_RS_, respiratory system resistance. ^ε^WT-Sal versus WT-Alt-Nuc, *P* < 0.0001; ^#^WT-Sal versus WT-Alt, *P* < 0.0001; ^§^WT-Sal versus *Cgas^–/–^*-Alt, *P* < 0.0001; *WT-Sal versus *Cgas^–/–^*-Alt-Nuc, *P =* 0.001; ^Ψ^WT-Alt-Nuc versus *Cgas^–/–^*-Alt-Nuc, *P* < 0.0001; ^Φ^WT-Alt versus WT-Alt-Nuc, *P* < 0.0001. (**B**–**E**) Quantification of eosinophils, neutrophils, lung inflammation, and mucus production in WT and *Cgas^–/–^* mice treated with Alt or Alt-Nuc (*n* = 5). (Groups are color-coded as in **A**). BM, basement membrane. (**F**) AHR after Alt-Nuc and Sal exposure in WT and *ALR^–/–^* mice (*n* = 5). *WT-Sal versus WT-Alt-Nuc, *P* < 0.0001; ^δ^WT-Sal versus *ALR^–/–^*-Alt-Nuc, *P =* 0.0003; ^Ψ^WT-Alt-Nuc versus *ALR^–/–^*-Alt-Nuc, *P* < 0.0001. (**G**–**J**) Quantification of eosinophils, neutrophils, lung inflammation, and mucus production in WT and *ALR^–/–^* mice treated with Alt-Nuc (*n* = 5). (Groups are color-coded as in **F**). (**K**) AHR after Alt-Nuc, Alt and Sal exposure in *Sting*^fl/fl^ and *LysM*Cre:*Sting*^fl/fl^ mice (*n* = 5). ^ε^*Sting*^fl/fl^-Sal versus *Sting*^fl/fl^-Alt-Nuc, *P* < 0.0001; ^#^*Sting*^fl/fl^-Sal versus *Sting*^fl/fl^ -Alt, *P* < 0.0001; *Sting^fl/fl^-Sal versus *LysM*Cre:*Sting*^fl/fl^-Alt, *P* < 0.0001; ^Ψ^*Sting*^fl/fl^-Alt-Nuc versus *LysM*Cre:*Sting*^fl/fl^-Alt-Nuc, *P*<0.0001; ^§^*LysM*Cre:*Sting*^fl/fl^-Alt versus *LysM*Cre:*Sting*^fl/fl^-Alt-Nuc, *P =* 0.02. (**L**–**O**) Quantification of eosinophils, neutrophils, lung inflammation, and mucus production in *Sting*^fl/fl^ and *LysM*Cre:*Sting*^fl/fl^ mice treated with Alt-Nuc, Alt, or Sal (*n* = 5). (Groups are color-coded as in **K**). (**P**) AHR after Alt-Nuc, Alt and Sal exposure in *RORα*^fl/fl^ and *IL7R*Cre:*RORα*^fl/fl^ mice (*n* = 5). ^δ^*RORα*^fl/fl^-Sal versus *RORα*^fl/fl^-Alt-Nuc, *P* < 0.0001; ^#^*RORα*^fl/fl^-Sal versus *RORα*^fl/fl^-Alt, *P* < 0.0001; ^§^*RORα*^fl/fl^-Sal versus *IL7R*Cre:*RORα*^fl/fl^-Alt, *P* = 0.007; ^Ψ^*RORα*^fl/fl^-Alt-Nuc versus *IL7R*Cre:*RORα*^fl/fl^-Alt-Nuc, *P* < 0.0001; *RORα^fl/fl^-Alt versus *RORα*^fl/fl^-Alt-Nuc, *P* < 0.0001; ^Φ^*RORα*^fl/fl^-Alt versus *IL7R*Cre:*RORα*^fl/fl^-Alt, *P* = 0.01. (**Q**–**T**) Quantification of eosinophils, neutrophils, lung inflammation, and mucus production in *RORα*^fl/fl^ and *IL7R*Cre:*RORα*^fl/fl^ mice (*n* = 5) treated with Alt-Nuc, Alt, and Sal (groups are color-coded as in **P**). Two-way ANOVA with Tukey’s multiple comparisons test was used to determine the statistical significance between groups. Data are presented as mean ± SEM. *P < 0.05, **P < 0.01, ***P < 0.001 ****P < 0.0001.

Next, we examined the role of the upstream DNA sensor cGAS in Alt-Nuc– and Alt-induced asthma. *Cgas^–/–^* mice showed significant inhibition of AHR, eosinophil and neutrophil influx in the BAL, and airway inflammation and mucus production in the Alt-Nuc model (Figure 7, A–E). *Cgas^–/–^* mice displayed modest inhibition of AHR and remarkable inhibition of eosinophilic inflammation and mucus production in the Alt model, which was different from that observed in *Sting^–/–^* mice. The latter suggested that cGAS regulated type 2 inflammation independent of STING. Control Sal treatment did not have any apparent effect in *Cgas*^+/+^ and *Cgas^–/–^* mice. There is no exact mouse ortholog for the human *IFI16* gene. Several genes (namely, *Ifi202, Ifi203, Ifi204*, and *Ifi205*) are considered similar to human *IFI16*. To eliminate the contribution of these genes, we studied the ALR (Aim2-like locus region) KO strain, which lacks 13 Aims2 and Ifi-like genes (35). *ALR^–/–^* mice showed a significant inhibition of AHR, airway inflammation, mucus production, and neutrophilic, but not eosinophilic, influx (Figure 7, F–J). The inhibition of lung neutrophilic inflammation was further confirmed by immunostaining for neutrophil MPO (Supplemental Figure 9D).

### STING in myeloid lineage cells was important for neutrophilic-eosinophilic asthma.

To investigate the role of STING expressed in cells of monocyte/macrophage/myeloid lineage, we generated *LysMCre:Sting*^fl/fl^ mice and studied them in Alt-Nuc and Alt models. The genetic deletion of STING in cells from the myeloid lineage caused significant inhibition of AHR, BAL eosinophils and neutrophils, lung MPO, airway inflammation, and mucus production (Figure 7, K–O, and Supplemental Figure 9E) in the Alt-Nuc model but not in the Alt model.

### Type 2 innate lymphoid cells were essential for Alt-Nuc–induced neutrophilic-eosinophilic asthma.

Because type 2 innate lymphoid cells (ILC2s) were the other cell type that internalized Nuc, we examined their role in Alt-Nuc– and Alt-induced asthma. We generated *IL7RCre:Rorα*^fl/fl^ mice (36) and subjected them to the Alt-Nuc and Alt protocols (Figure 2A). ILC2-deficient mice had reduced AHR, near-complete inhibition of inflammatory leukocytes (eosinophils, neutrophils, and lymphocytes) in BAL, and absent airway inflammation and mucus production (Figure 7, P and Q) in the Alt-Nuc model and, expectedly, in the Alt model. The results suggested that ILC2s were essential for Alt-Nuc–induced neutrophilic-eosinophilic asthma.

### The TNF-α–TNFR2–ICAM1 axis was critical for neutrophilic-eosinophilic asthma.

TNF-α is an important effector cytokine of the STING pathway (9). We recently reported on a TNFR2-expressing subtype of human ILCs (37). Another group reported that TNFR2 was essential for ILC2 survival, type 2 cytokine production, and generation of allergen-induced asthma in mice (38). Endothelial TNFR2 was critical for neutrophil transmigration (39). We found that Alt, but not Nuc, induced TNFR2 on lung cells from the mouse models (Supplemental Figure 10, A and B). The lung tissue from fatal asthma showed heightened TNFR2 staining of the blood vessel near macrophages (Supplemental Figure 10, C and D).

**Figure 8:**
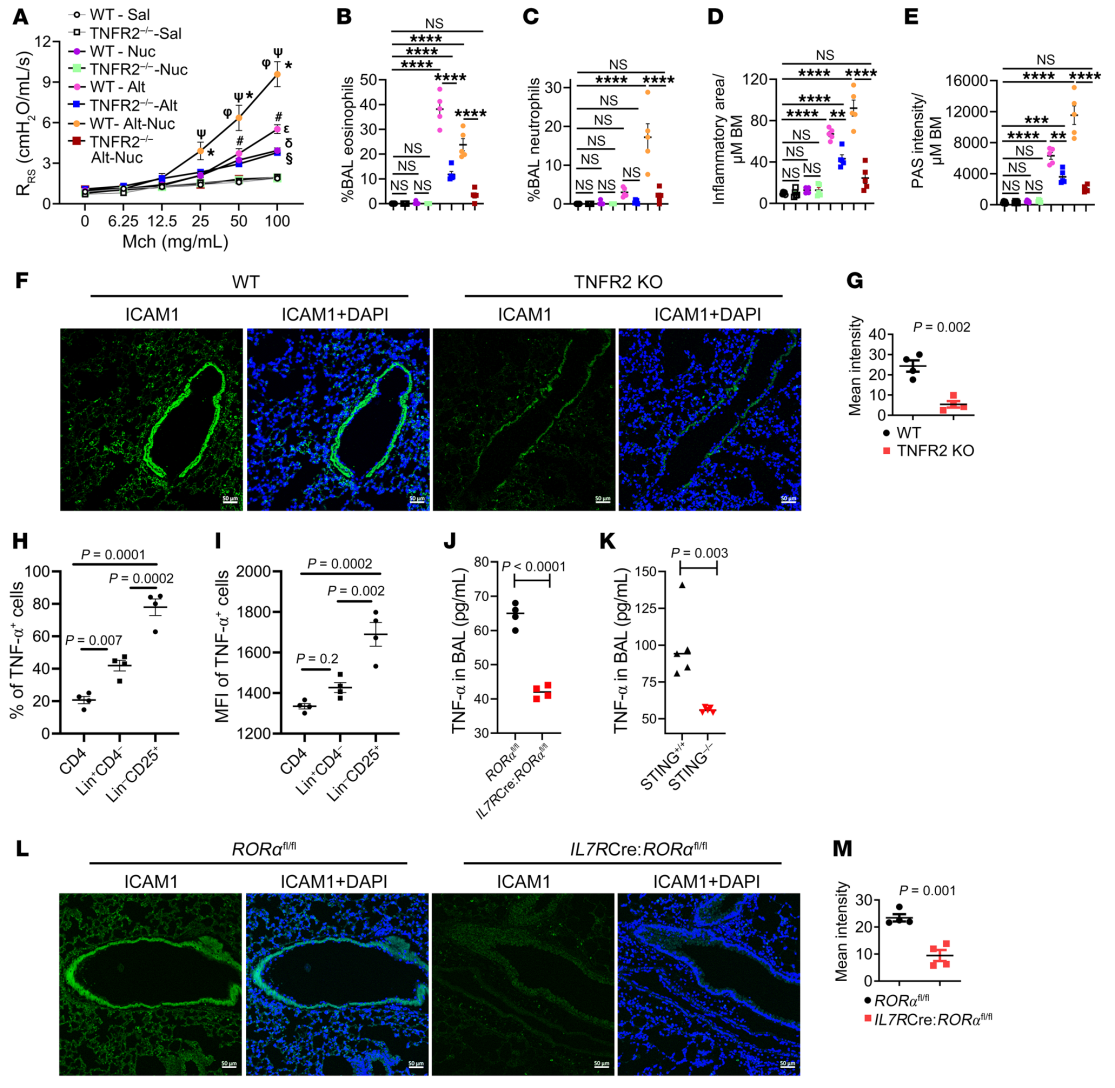
Role of TNF-α and TNFR2 in Alt-Nuc–induced asthma. (**A**) AHR following Alt-Nuc, Alt, Nuc and Sal exposure in WT and *Tnfr2^–/–^* mice as measured by flexiVent. R_RS_, respiratory system resistance. ^δ^WT-Sal versus WT-Nuc, *P* < 0.0001; ^ε^WT-Sal versus WT-Alt, *P* < 0.0001; ^§^WT-Sal versus *Tnfr2^–/–^*-Alt, *P* < 0.0001; *WT-Sal versus WT-Alt-Nuc, *P* < 0.0001; ^#^WT-Nuc versus WT-Alt, *P* = 0.006; ^φ^WT-Alt versus WT-Alt-Nuc, *P* < 0.0001; ^Ψ^WT-Alt-Nuc versus *Tnfr2^–/–^*-Alt-Nuc, *P* < 0.0001. (*n* = 5; 2-way ANOVA with Tukey’s multiple comparisons test). (**B**–**E**) Quantification of eosinophils and neutrophils in BAL, morphometric quantification of lung inflammation and mucus production in WT and *Tnfr2^–/–^* mice treated with Alt-Nuc, Alt, Nuc, or Sal (groups are color-coded as in **A**). (*n* = 5; 2-way ANOVA with Tukey’s multiple comparisons test). BM, basement membrane. (**F**) ICAM1 expression in the lung tissue of WT and *Tnfr2^–/–^* mice (*n* = 4) treated with Alt-Nuc. (**G**) Quantification of ICAM1 staining intensity in the lung tissue from WT and *Tnfr2^–/–^* mice treated with Alt-Nuc. Comparison made by Student’s 2-tailed *t* test. Scale bar, 50 μm. (**H** and **I**) Comparison of TNF-α^+^ lung hematopoietic cells (frequency and MFI) from the Alt-Nuc model (*n* = 4). (**J** and **K**) TNF-α (ELISA) in BAL from ILC2 KO (*IL7R*Cre:*Rorα*^fl/fl^) and *Sting^–/–^* mice (*n* = 4). (**L** and **M**) ICAM1 expression (immunostaining and mean intensity quantification) in the lung tissue from Alt-Nuc–treated *IL7R*Cre:*Rorα*^fl/fl^ and *Rorα*^fl/fl^ mice (*n* = 4). Scale bar, 50 μm. Comparison made by Student’s 2-tailed *t* test. Data are presented as mean ± SEM. *P < 0.05, **P < 0.01, ***P < 0.001, ****P < 0.0001.

To test the role of TNFR2, we studied *Tnfr2* (*Tnfrsf1b*)^+/+^ and *Tnfr2^–/–^* mice in our original Sal, Alt, Nuc, and Alt-Nuc models. Sal did not have any effect in WT and KO strains (Figure 8, A–E). As expected, Nuc caused mild AHR in *Tnfr2^+/+^* mice, which was absent in *Tnfr2^–/–^* mice. The KO mice had a significant inhibition of AHR, eosinophilic and neutrophilic influx in BAL, and airway inflammation and mucus production in both Alt and Alt-Nuc models; however, the effect was more pronounced in the Alt-Nuc model. An anti–TNFR2 Ab pretreatment significantly inhibited airway inflammation, mucus production, and the influx of neutrophils, but not eosinophils, into the airways in the Alt-Nuc model (Supplemental Figure 10, E–I). The results suggested the TNFα-TNFR2 axis was essential for neutrophilic-eosinophilic asthma in the Alt-Nuc model and eosinophilic asthma in the Alt model.

One of the mechanisms by which TNF-α induces inflammation is the induction of ICAM1 (39). We examined ICAM1 expression in *Tnfr2^+/+^* and *Tnfr2^–/–^* mice from the Alt-Nuc model. ICAM1 was largely absent in the lung tissue from *Tnfr2^–/–^* mice (Figure 8, F and G), confirming an essential role for TNFR2 in induction of ICAM1 and neutrophilic-eosinophilic inflammation.

### ILC2s were critical for production of TNF-α and induction of ICAM1.

ILC2s are known for regulation of type 2 eosinophilic inflammation. The absence of neutrophilic inflammation in ILC2 KO mice (Figure 7R) was a surprise. We investigated the mechanism of this unexpected finding and quantified TNF-α–producing lung cells from the Alt-Nuc model. About 78% of ILC2s (lin^–^CD25^+^) were positive for TNF-α as compared with 40% of lin^+^CD4^–^ (likely includes macrophages) and 20% of CD4^+^ cells (Figure 8, H and I, and Supplemental Figure 11, A–G). In contrast, only 12% of ILC2s were positive for TNF-α in the Alt-alone model of asthma (Supplemental Figure 11H). The gating strategy and the frequency of dual IL-5^+^ TNF-α^+^ ILCs are shown in Supplemental Figure 11, I–O. The results suggest Nuc converted the ILC2 from a low TNF-α–producing cell to a high TNF-α–producing cell. This is supported by a 36% reduction in TNF-α in BAL from ILC2 KO mice from the Alt-Nuc model (Figure 8J). The results also suggest that ILC2s, which constitute less than 0.5% of lung CD45^+^ cells, contributed to 36% of the total TNF-α. In comparison, there was a 45% decline in BAL TNF-α in global STING KO mice from the Alt-Nuc model (Figure 8K).

To examine whether the ILC2-derived TNF-α was critical for ICAM1 induction, we immunostained ICAM1 in the lung tissue from ILC2 KO mice. There was a near-complete loss of ICAM1 expression in the lung from *IL7RCre:Rorα*^fl/fl^ as compared with *Rorα*^fl/fl^ mice (Figure 8, L and M). We believe that in the Alt-Nuc exposure milieu, ILC2s become a critical contributor of TNF-α production, induction of ICAM1 and neutrophilic inflammation.

## Discussion

Increased extracellular DNA was reported to be increased in sputum from patients with asthma and correlated with asthma severity (13) and NA (14). Little is known about the role of Nucs, their receptors, and intracellular effectors such as STING in asthma. In this article, we demonstrated that extracellular DNA and Nuc were elevated in BAL, sputum, and serum from patients with NA. BAL DNA correlated with the BAL neutrophil counts. BAL DNA was partially associated with neutrophil MPO, suggesting neutrophilic origin of the DNA. Patients with NA had elevated DNA sensors, especially cytosol-translocated IFI16 and phosphorylated STING in bronchial biopsy specimens and BAL cells, which provided ex vivo evidence for DNA sensor activation. DNA/Nuc were noninflammatory when administered i.n. to mice. Coadministration with Alt unmasked the pro-neutrophilic and antieosinophilic inflammatory properties of DNA/Nuc and resulted in mixed neutrophilic-eosinophilic inflammation and other features of asthma. This phenotype was absent in mice deficient in cGAS, the *ALR* locus containing the IFI16 mouse ortholog IFI204, and STING, suggesting a critical role for the foregoing DNA sensors in this form of asthma. Increased extracellular IFI16 and the presence of anti-IFI16 autoantibodies have been reported in several rheumatological disorders including, Sjögren’s syndrome (40). The patients with NA did not have elevated levels of antinuclear Ab or antineutrophil cytoplasmic Ab (Table 1) or symptoms of autoimmune disorders.

One of the unexpected and important findings of the present study was that allergens abrogated the antiinflammatory properties of DNA and unmasked its proneutrophilic inflammatory activity. We identified multiple mechanisms. (a) Alt abrogated antiinflammatory and defensive gene induction by Nuc. (b) Alt (and other allergens) upregulated the receptor CCDC25, especially, CLEC2D, which facilitated internalization of DNA/Nuc, and, indirectly, activation of STING. (c) Alt activated the IL-33–ST2 pathway, which was essential for CLEC2D induction. (d) Alt caused cytosolic translocation of IFI16. (e) Alt activated ILC2s, which were important for TNF-α production. Despite their very low frequency (<0.1%) among CD45^+^ hematopoietic cells, ILC2s contributed to 36% of TNF-α in BAL. This source of TNF-α was essential for ICAM1 induction. (f) Alt upregulated TNFR2, which was critical for the phenotype. And (g) Alt synergized with Nuc in autophagy induction.

CLEC2D was reported to be a nonsignaling receptor for Nuc because it did not activate Syk, MAP kinases, and NF-κB (30). It augmented TLR9-mediated cytokine production. Our finding suggests that CLEC2D signaled through the STING signalosome. It bound and translocated Nucs to the cytosol and colocalized with STING. Cells with genetic deletion of CLEC2D were unable to phosphorylate STING, suggesting its critical role in activation of STING.

We showed that the TNF-α–TNFR2 axis was critical for neutrophilic-eosinophilic inflammation in our model. *Tnfr2^–/–^* mice were unable to upregulate ICAM1, induce leukocyte influx into the lung, and generate the asthma phenotype. Thus, we identified an Alt-Nuc–elicited STING–TNF-α–TNFR2–ICAM1 pathway that caused mixed neutrophilic-eosinophilic asthma. Nuc alone induced AHR but not inflammation. The foregoing phenotype mimics the pauci-inflammatory subtype of human asthma, which represents 20%–50% of all asthma (41, 42). The mechanism of human pauci-inflammatory asthma is poorly understood. Our transcriptomic analysis showed upregulation of genes involved in antimicrobial defense, inhibition of inflammation, and induction of smooth muscle hypertrophy, which could explain AHR and lack of inflammation in pauci-inflammatory asthma.

The frequency of cells that internalized Nuc was higher among macrophages than ILCs (Figure 4, C and D). This is expected because macrophages are professional phagocytes/scavenger cells. Two lines of evidence suggested the ILC2 contribution was higher than that of macrophages in the Alt-Nuc–induced phenotype. The frequency (percentage, but not absolute number) of TNF-α plus ILC2s was higher than that of TNF-α^+^Lin^+^CD4^–^ cells (78% vs. 40%). We speculate that a fraction of Lin^+^CD4^–^ cells were macrophages. More importantly, ILC2 KO (*IL7RCre:Rorα*^fl/fl^) mice showed a near-complete inhibition of eosinophilic and neutrophilic influx into the BAL, lung inflammation, and mucus production (Figure 7, Q–T). In comparison, the deletion of STING, the main effector of the Alt-Nuc phenotype, from macrophages did not completely inhibit eosinophilic influx, lung inflammation, and mucus production (Figure 7, L–O).

Based on our results, we speculate that intermittent infection (subclinical or overt) and/or exposure to environmental toxicants periodically increase airway Nuc from NETs and extracellular traps from other cells (e.g., macrophages), which superimpose neutrophilic inflammation on an existing eosinophilic inflammation in asthma. The induction of NET-driven neutrophilic inflammation depends on activation of STING. The STING gene is polymorphic and 1 set of polymorphisms is quite common in the population (43). The so-called HAQ haplotype of STING has significantly attenuated function, which may explain why not all patients with asthma experience sustained neutrophilic inflammation despite having intermittent infection and pollution exposure.

## Methods

### Sex as a biological variable.

We studied specimens from female and male participants in experiments involving human samples. We performed animal experiments in female mice. In select experiments involving macrophages, we additionally performed experiments in male mice. We did not perform any ILC-related experiments in male mice, because their ILC number was reduced by 50% as compared with female mice.

### Study participants.

Patients with asthma were recruited to the outpatient clinics at National Jewish Health (NJH) and were defined according to the ATS consensus definition. Bronchoscopy and BAL were performed along with clinical workup. None of the DC patients met the ATS diagnostic criteria for asthma. The demographic and diagnostic characteristics of healthy donors and patients with asthma are detailed in Table 1.

### Mice.

Our study examined female and male mice and similar findings are reported for both sexes. Female C57BL/6 mice, aged 6–8 weeks were purchased from The Jackson Laboratory. *Sting*^fl/fl^ mice and *Sting^–/–^* mice were provided by John Cambier (University of Colorado). *ALR^–/–^* mice were a gift from David Stetson (University of Washington). *Cgas^–/–^* (B6(C)-*Cgas^tm1d(EUCOMM)Hmgu^/*J) and *Tnfr*2*^–/–^* mice (B6.129S7-*Tnfrsf1b^tm1Imx^/J*) were purchased from The Jackson Laboratory. In addition, mice with Cre recombinase-ERT2 fusion gene driven by *LysM* promotor and *Sting*^fl/fl^ mice were crossed to generate mice carrying *LysM*Cre/ERT:*Sting*^fl/fl^. Macrophage-specific deletion of *Sting* was achieved via intraperitoneal injection of 100 μL of 10 mg/mL tamoxifen (Sigma; T5648) for 5 consecutive days into 4-week-old *LysM*Cre/ERT:*Sting*^fl/fl^ female mice. Tamoxifen-treated mice were rested at least 3 weeks before use in experiments. In addition, 8-week-old female ILC-depleted mice (*IL7R*Cre:*RORα*^fl/fl^ mice) were obtained from Kita Lab and used in experiments.

### Asthma model.

For the generation of the mixed neutrophilic-eosinophilic asthma model, 8-week-old female mice were challenged i.n. daily with a combination of Alt (10 μg) and Nuc (10 μg) for 5 days, rested for 2 days after the last challenge, and then examined for AHR, lung-infiltrating immune cells, and lung inflammation. The *Alternaria* allergen extract (catalog XPM1D3A25) and *Aspergillus* extract (catalog XPM3D3A4) were from Greer Labs. Nucs (catalog 52039) and histones (catalog 52065) were from BPS Bioscience, Inc. Mouse lung–derived DNA (catalog HG601) was from Zyagen, Inc.

### AHR measurement.

AHR was assessed on a flexiVent v6.2 rodent ventilator (Scireq). Briefly, 2 days after the last i.n. challenge, mice were anesthetized with ketamine (180 mg/kg), xylazine (9 mg/kg), and acepromazine (4 mg/kg). Tracheotomy was performed and mice were attached via an 18-gauge cannula to small-animal ventilator with computer-controlled piston. Resistance measurements were obtained at baseline (Sal) and after increasing concentrations of methacholine (MCh). Resistance measurements were obtained after nebulization of each concentration of MCh. Airway resistance was expressed as total respiratory system resistance. Data are presented as mean ± SEM. Multiple groups were compared using 2-way ANOVA followed by Tukey’s post hoc multiple comparison test.

### Histology and morphometric analysis.

Formalin-fixed and paraffin-embedded lung tissue sections (5 μm) were stained with H&E for morphometric analysis and periodic acid–Schiff (PAS) for mucin staining and mounted using permount medium (Thermo Fisher Scientific). Images were acquired on a Nikon Eclipse TE2000-U microscope using ×20 dry lenses through a Diagnostics Instruments camera model 4.2 using Spot software 5.0. Inflammation and PAS staining intensity was quantified with Metamorph image acquisition and analysis software (Molecular Devices), as reported previously (44). Airway inflammation was measured as the ratio of the total area of peribronchial and perivascular inflammatory infiltrates over the perimeter of the associated basement membrane obtained from a minimum of 5 airways per mouse and 5 mice per group.

### IHC.

Formalin-fixed and paraffin-embedded lung tissue sections (5 μm) were deparaffinized with Citisolv, rehydrated in graded alcohol series, and further rehydrated in PBS. Heat-induced antigen retrieval was done by microwaving the sections for 10 minutes in 10 mM citrate buffer (pH 6.0) (Sigma; C9999). Sections were blocked with blocking buffer (5% BSA in PBS) for 1 hour at room temperature (RT) and were incubated overnight in a humid chamber at 4°C in primary Abs diluted in blocking buffer; followed by 3 washes with PBS. Appropriate fluorescent conjugated secondary Abs (Molecular Probes) diluted in blocking buffer (1:1,000) were added to the sections for 1 hour at RT in dark, followed by 3 PBS washes, and mounted using Vectashield antifade reagent containing DAPI (Vector Labs; H-2000). The details of primary Abs and the dilutions at which they were used for IHC samples in paraffin are given in Table 2. Images were acquired using a Zeiss LSM 700 confocal microscope with a ×40 oil immersion objective lens. Images were analyzed and processed using Zen Blue software (Carl Zeiss) and further processed using Adobe Photoshop. Pearson correlation coefficients for colocalization were calculated by Zen Blue software.

### IHC.

Human alveolar macrophages (or BAL macrophages) were grown on coverslips, and we followed the same staining procedure as described above except that coverslips were not subjected to deparaffinization, rehydration with graded alcohol series, or heat-induced antigen retrieval steps. Instead, cells were permeabilized in PBS containing 0.5% TritonX-100 and 0.05% Tween-20 for 5 minutes before the staining procedure. Images were acquired using a Zeiss LSM 700 confocal microscope with a ×63 oil immersion objective lens and analyzed as above.

### Western blotting.

Briefly, cells were washed with ice-cold PBS and lysed in RIPA buffer (Thermo Fisher Scientific; 89900) with protease and phosphatase inhibitor cocktail (Sigma; PPC2020) before estimation of the protein quantity by bicinchoninic acid protein assay kit (Pierce; 23225). Cytosolic and nuclear fractionation was done following the protocols associated with the Nuclear/Cytosol Extraction Kit (Abcam; ab289882). Equal amounts of protein were loaded on SDS-PAGE gel and then transferred onto nitrocellulose membrane (GE; 10600001). Blots were subsequently blocked with 5% Blotto (Santa Cruz Biotechnology; sc-2325) in TBS-T for 1 hour at RT, followed by overnight incubation with primary Abs. After 3 washes with TBS-T (5 minutes each), blots were incubated in HRP-conjugated secondary Abs for 1 hour at RT, followed by 3 washes with TBS-T. Pierce ECL Western blotting substrate (catalog 32209) was used to visualize bands, and the bands were quantified using the ImageJ software. The primary Abs used for Western blotting are listed in Table 2.

### FCM.

Briefly, lungs were dissected, minced, and incubated in RPMI1640 medium (Corning; 10-040-CV) supplemented with 10% FBS (Gibco; A56708-01), 1× penicillin/streptomycin (Corning; 30-002-C1), and 200 μg/mL Liberase (Roche; 5401127001) for 45 minutes at RT and treated with ACK lysis buffer (Quality Biological; 118-156-101) to remove RBCs. Single cells from the lung were suspended in PBS and stained with the Zombie Aqua Fixable viability kit (Biolegend; 423102) to remove dead cells. After washing, cells were resuspended in FACS buffer (PBS containing 0.5% BSA), blocked with anti-CD16/CD32 Abs (Biolegend; 101320), and stained with fluorochrome-labelled mAbs against cell-surface markers for 30 minutes at 4°C. For intracellular staining, the Fixation/Permeabilization solution kit (BD; 554714) was used according to the manufacturer’s protocol. Details of the fluorochrome-tagged Abs are listed in Table 2.

For staining of human cells from BAL and PBMCs, single-cell suspensions were stained with the Abs (Table 2). Data were collected on an LSRFortessa X- 20 device (BD Bioscience) and analyzed by FlowJo, version 10.2, software.

### DNA picogreen assay.

The concentration of dsDNA in human BAL and serum samples was measured using Quant-iT PicoGreen dsDNA reagent (Invitrogen; P11496), following the manufacturer’s protocol. We also measured the concentration of DNA in 10 μg of allergens (dry weight) (Alt, Asp, and dust mites) (Greer Labs; XPM1D3A25, XPM3D3A4, and XPB81D3A25, respectively) and 10 μg of PM_2.5_ (National Institute of Standards and Technology, US Department of Commerce).

### Measurement of Nuc-bound DNA.

Histone-associated DNA in BALF from patients with NA or N-NA and from the DC group was assessed by photometric enzyme immunoassay (cell death detection ELISAPLUS kit; Roche, 11920685001). The assay is based on the quantitative sandwich enzyme immunoassay principle using mouse mAbs directed against DNA and histones. This allows the specific determination of mono- and oligonucleosomes in the BALF. Briefly, BALF samples were placed into a streptavidin-coated microplate and incubated with a mixture of anti–histone-biotin and anti–DNA-peroxidase. While in incubation, the histone component of the Nucs was captured by the anti–histone-biotin Ab, and the DNA component of the Nucs was detected by an anti–DNA-peroxidase Ab. After removal of the unbound Abs, the amount of peroxidase present in the immunocomplex was photometrically determined with ABTS (2,2′-azinodi-(3 ethylbenzthiazolinesulfonic acid)) as the substrate.

### ELISA.

Briefly, after measuring AHR by flexiVent, mouse lung BALF was collected by washing the lungs with 1 mL of PBS, and the cytokines TNF-α (R&D Systems; DY410), GDF15 (R&D Systems; DY6385) and BPIFA1 (antibodies-online; ABIN6954161) were estimated in the BALF by following manufacturer’s instructions. Cytokines IL-1β (catalog DY201), IL-6 (catalog DY206), IP10 (catalog DY266), IL-17A (catalog DY317), CXCL9 (catalog DY392), and IFN-β (catalog DY814) from human BAL, serum, and sputum samples, and from human macrophage cell culture supernatants were similarly measured with ELISA Duoset kits (R&D Systems) following manufacturer’s instructions.

### Cytokine array.

Briefly, human BAL macrophages were cultured in the absence or presence of Nucs for the indicated time points. Supernatants were collected and cytokine array was performed using Human Cytokine Array C5 (Raybiotech; AAH-CYT-5-8) according to the manufacturer’s instructions. Similarly, mouse BALF samples were collected and cytokine array was performed using Mouse Cytokine array C3 (catalog AAM-CYT-3-8). Spot intensities were calculated using ImageJ software and normalized to the reference spots. Protein expression levels were described in arbitrary units as depicted in the graph (Figure 2, P and Q).

### Real-time PCR.

DNA-free total RNA was prepared using the RNeasy Plus Mini Kit (Qiagen; 74134) and was reverse transcribed into cDNA with the Superscript III first-strand synthesis kit according to manufacturer’s instructions. Gene-specific PCR products were amplified by using qPCR SYBR Green Rox mix. The copy numbers of *Bpifa1*, *Bpifb1*, *Ddit1*, *Ltf*, and *Gdf15* were normalized to that of *Gapdh* by using the comparative Ct method. The following primers were used: *Bpifa1*: forward 5′-ATCCAGGGCAAGGTATGTCC-3′ and reverse 5′-TCTGTGAGCCATGGGAGACT-3′; *Bpifb1*: forward 5′-CAGCCACCCCGAGGTCCTA-3′ and reverse 5′-GAGGGTGATAATCCACGGGC-3′; *Ltf*: forward 5′-CCAGGCCATTGTGACAAACAG -3′ and reverse 5′-CACGACTGCTACCGCATAGT-3′; *Ddit1*: forward 5′-GAAATCCGGCAGCGCCTA-3′ and reverse 5′-TAGCCACCGTCCAACAACTC-3′; *Gdf15*: forward 5′-CTCCAGGTTCTTGGGCTTCC-3′ and reverse 5′-CTGGAGGACAGACCGCT-3′; and *Gapdh*: forward 5′-CTTTGTCAAGCTCATTTCCTGG-3′ and reverse 5′-TCTTGCTCAGTGTCCTTGC-3′. Real-time PCR was performed with the ABI Prism 7000 Sequence Detection System (Applied Biosystems).

### CRISPR-Cas9–mediated knockdown of IFI16 and CLEC2D.

Briefly, blood monocyte–differentiated macrophages were electroporated by the Lonza transfection system with Ctrl single-guide RNA (sgRNA) or IFI16 and CLEC2D sgRNAs obtained from Sigma (30 pM each). Cells (5 × 10^4^) were resuspended in 100 μL of 4-D Nucleofector Solution (Lonza;# V4XP-3024) and mixed with Cas9 ribonucleoproteins, followed by electroporation (1005 V, 35 ms, 2 pulses) according to the manufacturer’s instructions. After electroporation, cells were plated in 12-well plates in the presence of 10 ng/mL macrophage CSF (Peprotech; 300-25) for 48 hours. After 48 hours, the cells were treated with allergen and Nucs (5 μg each), and supernatants were collected and cytokine secretion was determined by ELISA following manufacturer’s instructions.

### RNA-Seq.

Lung tissue samples were collected from a total of 12 mice (*n* = 3 mice per group) treated with Sal, Nuc, Alt, and Alt-Nuc. Total RNA was isolated using the standard RNA isolation kit from Qiagen (catalog 74134). The isolated total RNA was processed for next-generation sequencing library construction as developed in the NJH Genomics Facility for analysis with an Illumina NovaSeq 6000. A Kapa Biosystems Hyper mRNA-Seq kit for whole-transcriptome libraries was used to primarily target all polyA RNA. Briefly, library construction started from isolation of total RNA species, followed by mRNA (poly-A) isolation, first and second-strand cDNA synthesis, adaptor ligation, amplification, and cluster generation. Once validated, the libraries were sequenced as barcoded-pooled samples and run on the NovaSeq 6000 on a S4 flow cell with 2 × 150 bp sequencing chemistry. After the library build, genes with not more than 1 count per million reads in fewer than 3 mice were removed. We used DEG-Seq to identify differentially expressed genes among the Sal, Nuc, Alt, Alt-Nuc groups. A *P* value < 0.05 and log2 fold change >2 were selected as significant difference thresholds.

### Gene Ontology and pathway enrichment analysis.

Gene Ontology functional annotation and pathway analysis were performed on differential expressed genes using the PANTHER database and classification system (pantherdb.org).

### Statistics.

The 2-tailed Student’s *t* test (parametric) was used for the comparisons between 2 groups or paired *t* test. Multiple groups were compared by ANOVA followed by Tukey’s post hoc multiple comparison (parametric). Human samples were analyzed by Mann-Whitney U (nonparametric) test. Correlations were determined by Pearson’s correlation coefficient (parametric) and Spearman’s rank correlation (nonparametric) tests. All experiments were performed at least in triplicates and the results are presented as mean ± SEM. *P* < 0.05 was considered statistically significant. Statistical analysis was performed with GraphPad Prism 7 software (GraphPad Software).

### Study approval.

All animal experiments were approved by the IACUC committee of NJH (protocol AS2614-11-23). Protocols for blood and BAL studies from patients with asthma and those in the DC group were approved by the IRB (protocols HS-1700-528, HS-3153, and HS-2918) and were conducted in accordance with the principles of good clinical practice. All patients provided written informed consent.

### Data availability.

Raw sequencing data are deposited in the NCBI Gene Expression Omnibus (GEO) database (GEO GSE297626). All data are available in the main text or the supplemental materials. Values for data points in graphs are reported in the Supporting Data Values file.

## Author contributions

RA conceived and designed experiments, wrote the manuscript, and secured funding. A. Sripada designed experiments, analyzed and prepared data, and wrote up the methods. DV, RV, KS, CK, and A. Sahu performed some of the experiments, and MV and MP assisted in experiments. VG and LM performed bronchoscopy. BV and CM performed statistical analysis. RJM, MMG, JC, and AG critically reviewed the manuscript.

## Supplementary Material

Supplemental data

Unedited blot and gel images

Supporting data values

## Figures and Tables

**Table 1:**
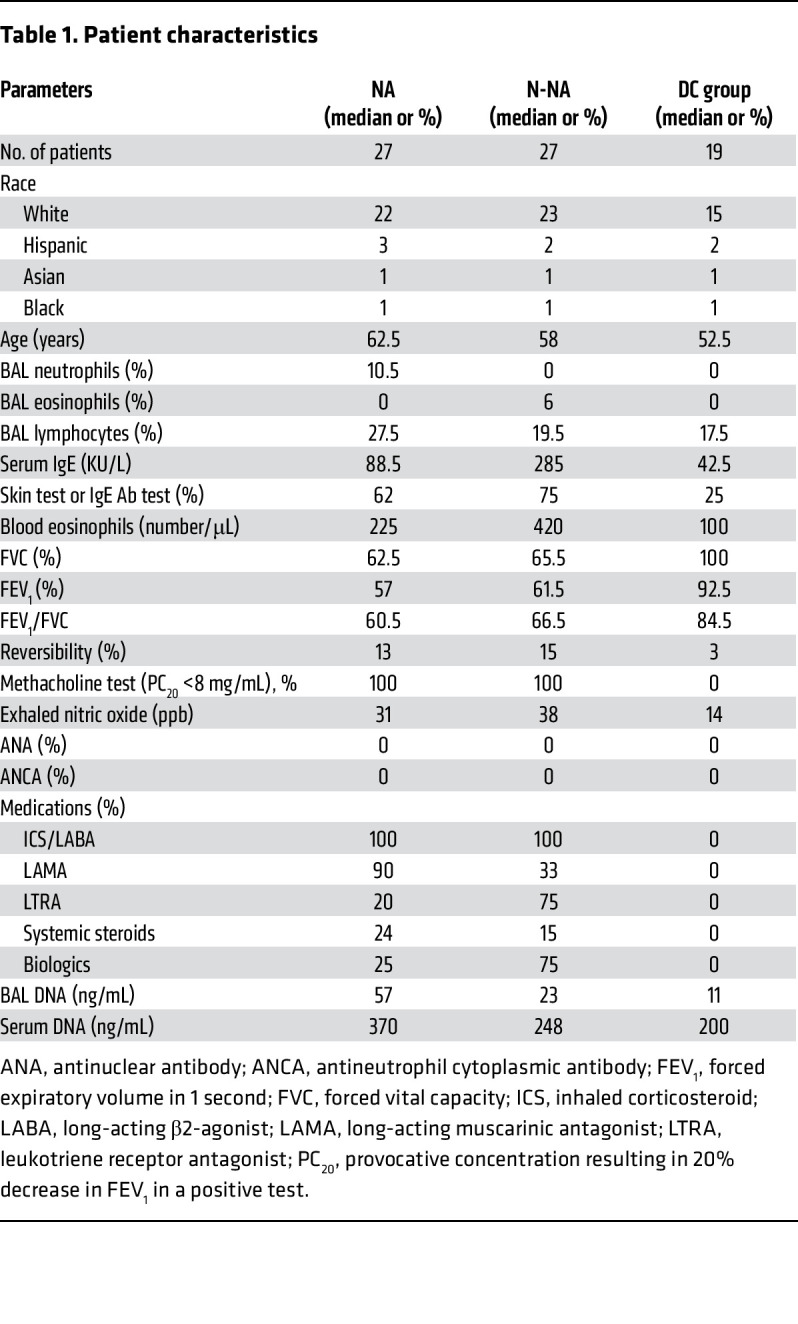
Patient characteristics

**Table 2:**
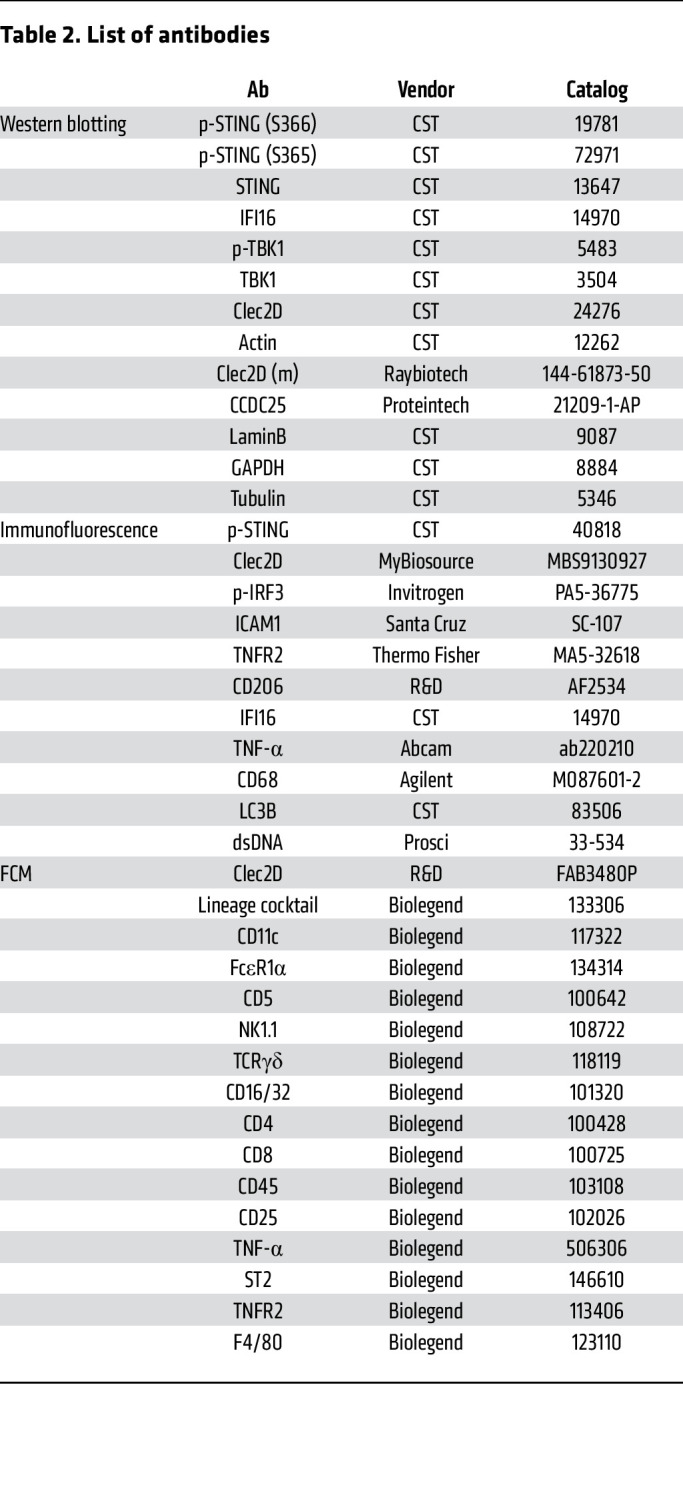
List of antibodies

## Version 1 06/17/2025

In-Press Preview

## Version 2 08/15/2025

Electronic publication

## References

[1] Wenzel SE (2021). Severe adult asthmas: integrating clinical features, biology, and therapeutics to improve outcomes. Am J Respir Crit Care Med.

[2] Bacharier LB, Jackson DJ (2023). Biologics in the treatment of asthma in children and adolescents. J Allergy Clin Immunol.

[3] Murphy RC (2021). Management strategies to reduce exacerbations in non-T2 asthma. J Allergy Clin Immunol Pract.

[4] Alam R (2017). Airway and serum biochemical correlates of refractory neutrophilic asthma. J Allergy Clin Immunol.

[5] Steinke JW (2021). Bronchoalveolar lavage cytokine patterns in children with severe neutrophilic and paucigranulocytic asthma. J Allergy Clin Immunol.

[6] Gibson PG (2017). Effect of azithromycin on asthma exacerbations and quality of life in adults with persistent uncontrolled asthma (AMAZES): a randomised, double-blind, placebo-controlled trial. Lancet.

[7] Altman MC (2019). Transcriptome networks identify mechanisms of viral and nonviral asthma exacerbations in children. Nat Immunol.

[8] Nagler M (2018). Extracellular DNA in natural environments: features, relevance and applications. Appl Microbiol Biotechnol.

[9] Dou Z (2017). Cytoplasmic chromatin triggers inflammation in senescence and cancer. Nature.

[10] Lan YY (2019). Extranuclear DNA accumulates in aged cells and contributes to senescence and inflammation. Aging Cell.

[11] Han DSC (2020). The biology of cell-free DNA fragmentation and the roles of DNASE1, DNASE1L3, and DFFB. Am J Hum Genet.

[12] Giordano AMS (2022). DNA damage contributes to neurotoxic inflammation in Aicardi-Goutières syndrome astrocytes. J Exp Med.

[13] Lachowicz-Scroggins ME (2019). Extracellular DNA, neutrophil extracellular traps, and inflammasome activation in severe asthma. Am J Respir Crit Care Med.

[14] Abdo M (2021). Raised sputum extracellular DNA confers lung function impairment and poor symptom control in an exacerbation-susceptible phenotype of neutrophilic asthma. Respir Res.

[15] Chen Q (2016). Regulation and function of the cGAS-STING pathway of cytosolic DNA sensing. Nat Immunol.

[16] Guntur VP (2022). Refractory neutrophilic asthma and ciliary genes. J Allergy Clin Immunol.

[17] Thaikoottathil JV (2012). SPLUNC1 deficiency enhances airway eosinophilic inflammation in mice. Am J Respir Cell Mol Biol.

[18] Kruzel ML (2006). Lactoferrin decreases pollen antigen-induced allergic airway inflammation in a murine model of asthma. Immunology.

[19] Niwa S (2012). KIF19A is a microtubule-depolymerizing kinesin for ciliary length control. Dev Cell.

[20] Hwang JY (2023). LRRC23 truncation impairs radial spoke 3 head assembly and sperm motility underlying male infertility. Elife.

[21] Spaich S (2012). F-box and leucine-rich repeat protein 22 is a cardiac-enriched F-box protein that regulates sarcomeric protein turnover and is essential for maintenance of contractile function in vivo. Circ Res.

[22] Nishida W (2002). A triad of serum response factor and the GATA and NK families governs the transcription of smooth and cardiac muscle genes. J Biol Chem.

[23] Zhang C (2022). Small proline-rich proteins (SPRRs) are epidermally produced antimicrobial proteins that defend the cutaneous barrier by direct bacterial membrane disruption. Elife.

[24] Sutherland TE (2014). Chitinase-like proteins promote IL-17-mediated neutrophilia in a tradeoff between nematode killing and host damage. Nat Immunol.

[25] Chiba Y (2018). Augmented Pla2g4c/Ptgs2/Hpgds axis in bronchial smooth muscle tissues of experimental asthma. PLoS One.

[26] Hoch T (2022). Multiplexed imaging mass cytometry of the chemokine milieus in melanoma characterizes features of the response to immunotherapy. Sci Immunol.

[27] Lood C (2017). TLR7/8 activation in neutrophils impairs immune complex phagocytosis through shedding of FcgRIIA. J Exp Med.

[28] Ericson JA (2014). Gene expression during the generation and activation of mouse neutrophils: implication of novel functional and regulatory pathways. PLoS One.

[29] Yang L (2020). DNA of neutrophil extracellular traps promotes cancer metastasis via CCDC25. Nature.

[30] Lai JJ (2020). Immune sensing of cell death through recognition of histone sequences by C-type lectin-receptor-2d causes inflammation and tissue injury. Immunity.

[31] Srisomboon Y (2023). Allergen-induced DNA release by the airway epithelium amplifies type 2 immunity. J Allergy Clin Immunol.

[32] Gui X (2019). Autophagy induction via STING trafficking is a primordial function of the cGAS pathway. Nature.

[33] Jin L (2013). STING/MPYS mediates host defense against Listeria monocytogenes infection by regulating Ly6C(hi) monocyte migration. J Immunol.

[34] Haag SM (2018). Targeting STING with covalent small-molecule inhibitors. Nature.

[35] Gray EE (2016). The AIM2-like receptors are dispensable for the interferon response to intracellular DNA. Immunity.

[36] Ferreira ACF (2021). RORα is a critical checkpoint for T cell and ILC2 commitment in the embryonic thymus. Nat Immunol.

[37] Liu S (2020). Optimal identification of human conventional and nonconventional (CRTH2^-^IL7Rα^-^) ILC2s using additional surface markers. J Allergy Clin Immunol.

[38] Hurrell BP (2019). TNFR2 signaling enhances ILC2 survival, function, and induction of airway hyperreactivity. Cell Rep.

[39] Ren X (2023). Macrophage-endothelial cell crosstalk orchestrates neutrophil recruitment in inflamed mucosa. J Clin Invest.

[40] Antiochos B (2018). IFI16 filament formation in salivary epithelial cells shapes the anti-IFI16 immune response in Sjögren’s syndrome. JCI Insight.

[41] Wenzel S (2005). Severe asthma in adults. Am J Respir Crit Care Med.

[42] Teague WG (2019). Lung lavage granulocyte patterns and clinical phenotypes in children with severe, therapy-resistant asthma. J Allergy Clin Immunol Pract.

[43] Jin L (2011). Identification and characterization of a loss-of-function human MPYS variant. Genes Immun.

[44] Sripada A (2021). Sprouty2 positively regulates T cell function and airway inflammation through regulation of CSK and LCK kinases. PLoS Biol.

