# Supplementary material for: Allergens abrogate antiinflammatory DNA effects and unmask macrophage-driven neutrophilic asthma via ILC2/STING/TNF-α signaling
Source: J Clin Invest. 2025 Jun 17;135(16):e187907. doi: 10.1172/JCI187907 (PMC12352889; doi:10.1172/JCI187907)
Supplement: Supplemental data [file jci-135-187907-s047.pdf]

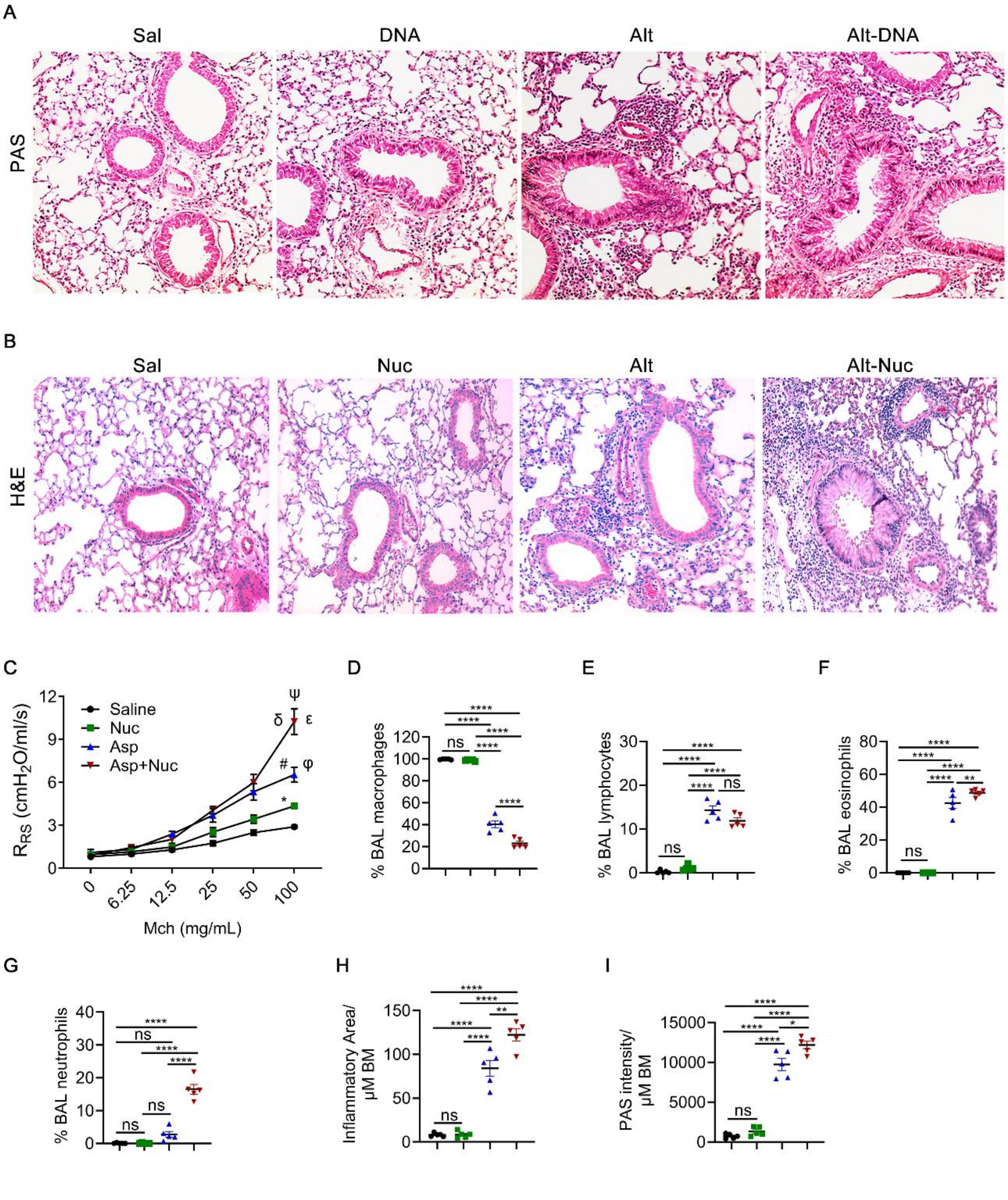


**Supplementary Fig. 1**. (**A and B**) Representative PAS and H&E staining of the lung tissue from Sal, Alt, Nuc and Alt+Nuc treated B6 mice as per **Fig. 2A**. (**C**) AHR following Saline, Nuc, Aspergillus (Asp) or Asp+Nuc exposure in B6 mice as measured by flexiVent. *(Sal vs Nuc) $P$=0.01; φ(Sal vs Asp) $P$<0.0001; ε(Sal vs Asp+Nuc) $P$<0.0001; #(Nuc vs Asp) $P$<0.0001; δ(Nuc vs Asp+Nuc) $P$<0.0001; ψ(Asp vs Asp+Nuc) $P$=0.002. . (n=5; 2-way ANOVA, Tukey's multiple comparisons test).

(**D-G**) Differential counts of BAL macrophages, lymphocytes, eosinophils and neutrophils. (**H and I**) Morphometric quantification of lung inflammation and mucus production following H&E and PAS staining of the lung tissue (groups were color-coded as in C). (n=5; 2-way ANOVA, Tukey's multiple comparisons test). Data are presented as mean+/-SEM, **P*<0.05, ***P*<0.01, ****P*<0.001 *****P*<0.0001, ns=not significant.

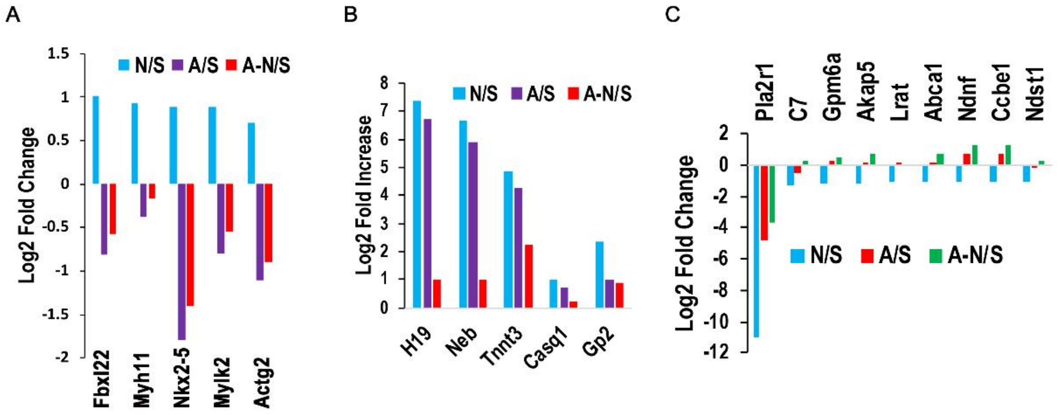


D

| PANTHER Overrepresentation Test, GO Ontology | |
|---|---|
| GO Biological Process | FDR (AN vs A) |
| Regulation of modification of postsynaptic actin cytoskeleton (GO:1905274) | 3.46E-02 |
| Activation of innate immune response (GO:0002218) | 9.11E-04 |
| Positive regulation of NIK/NF-kappaB signaling (GO:1901224) | 1.18E-04 |
| Cellular response to interferon-beta (GO:0035458) | 9.44E-03 |
| Body fluid secretion (GO:0007589) | 1.02E-02 |

**Supplementary Fig. 2**. Top Nuc up- and down-regulated genes in the lung from Nuc-treated mice and comparison with Sal, Alt and Alt-Nuc treated mice. (**A**) Genes that were selectively upregulated by Nuc but inhibited by Alt and Alt-Nuc. (**B**) Genes upregulated by all three treatment protocols--Nuc, Alt and Alt-Nuc. (**C**) Genes down-regulated by Nuc. (**D**) Top GO biological processes in Alt-Nuc vs Alt treatment groups.

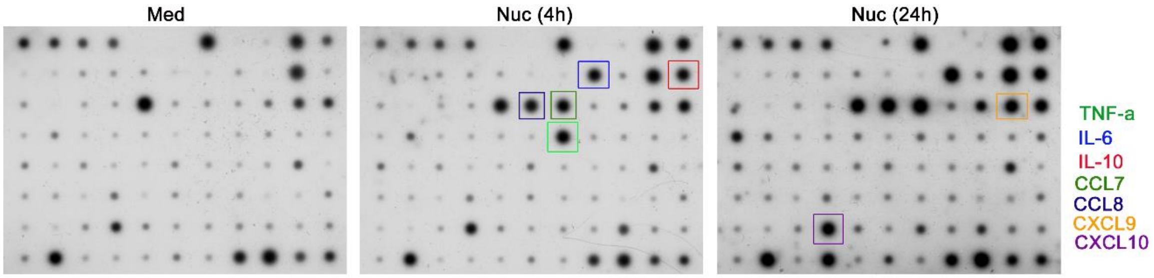


**Supplementary Fig. 3**. Nuc-induced cytokines/chemokines. Human BAL macrophages were cultured with medium (Med), and Nuc for 4 and 24 hr and the culture supernatant was assayed for 80 cytokines/chemokines using the human cytokine array C5 from RayBiotec. The cytokine/chemokine that was upregulated by Nuc is shown in color-coded square symbol.

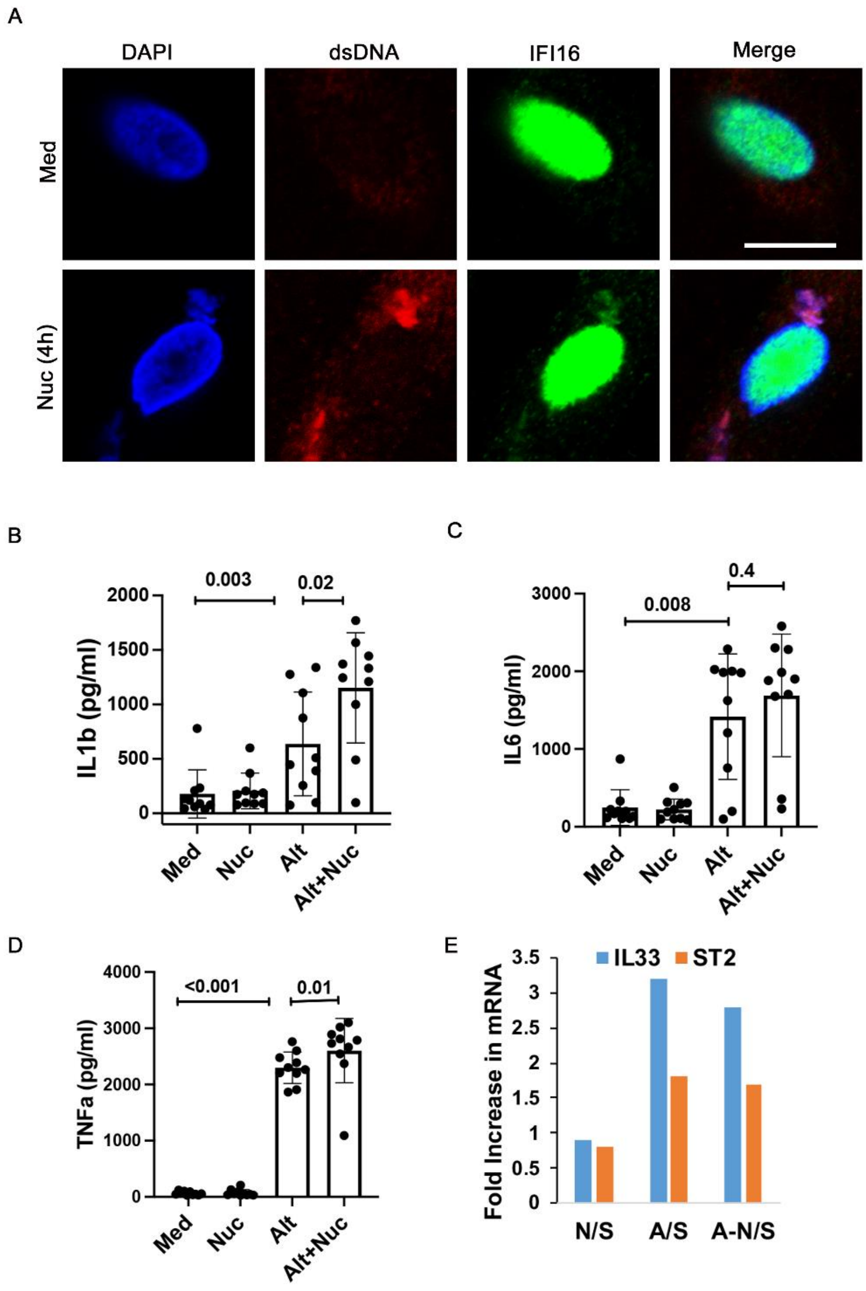


**Supplementary Fig. 4**. Nuc uptake and induction of cytokines. (**A**) Nuc uptake by human BAL macrophages. Macrophages were incubated with Nuc or medium for 4 hr, stained for cytosolic dsDNA (red) and IFI16 (green) and counterstained with DAPI. The anti-dsDNA antibody specifically detects cytosolic but not nuclear dsDNA. One representative image is shown. Scale bar, 5 µm. (**B-D**) Cytokine production by monocytes. Blood monocytes from asthma patients were cultured with. Medium, Nuc (10 µg/ml), Alt (10 µg/ml) and Alt + Nuc (10 µg/ml each) for 24 hr and

assayed for the indicated cytokines. Statistical analyses by Mann-Whitney U test. (**E**) The increase in mRNA for *il33* and *il1rl1* (ST2) in the lung from the study groups as compared to the Saline (S) group.

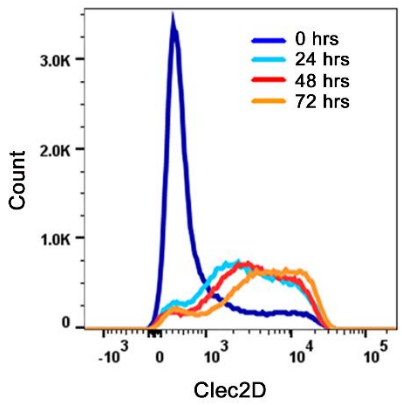


**Supplementary Fig. 5**. Flow cytometric measurement of CLEC2D following stimulation with Alt for 0, 24, 48, and 72 hrs (n=4).

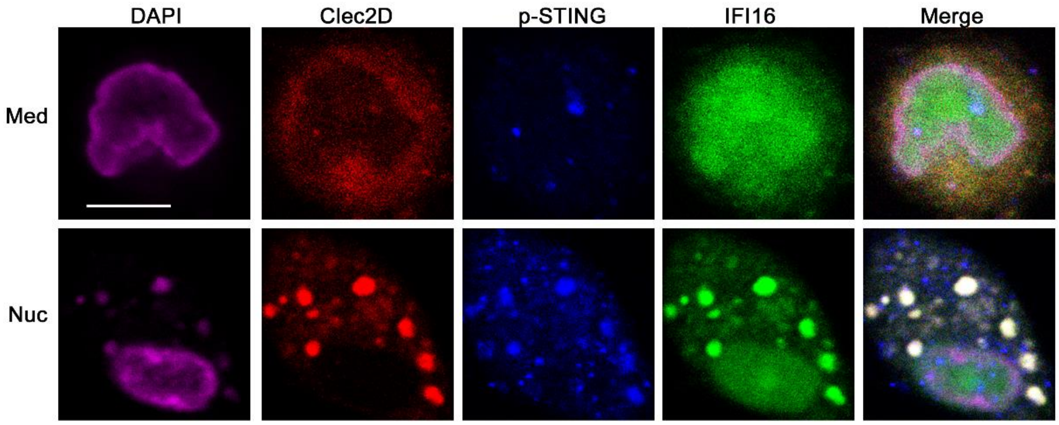


**Supplementary Fig. 6**. CLEC2D immunostaining. Triple immunostaining of human BAL macrophages for CLEC2D (red), p-STING (blue) and IFI16 (green) and counterstaining with DAPI (pink) following incubation with Nuc for 4 hr. Co-localization (white) of internalized CLEC2D with p-STING and cytosolic IFI16 is shown in the merged image (n=3). Scale bar, 5 μm.

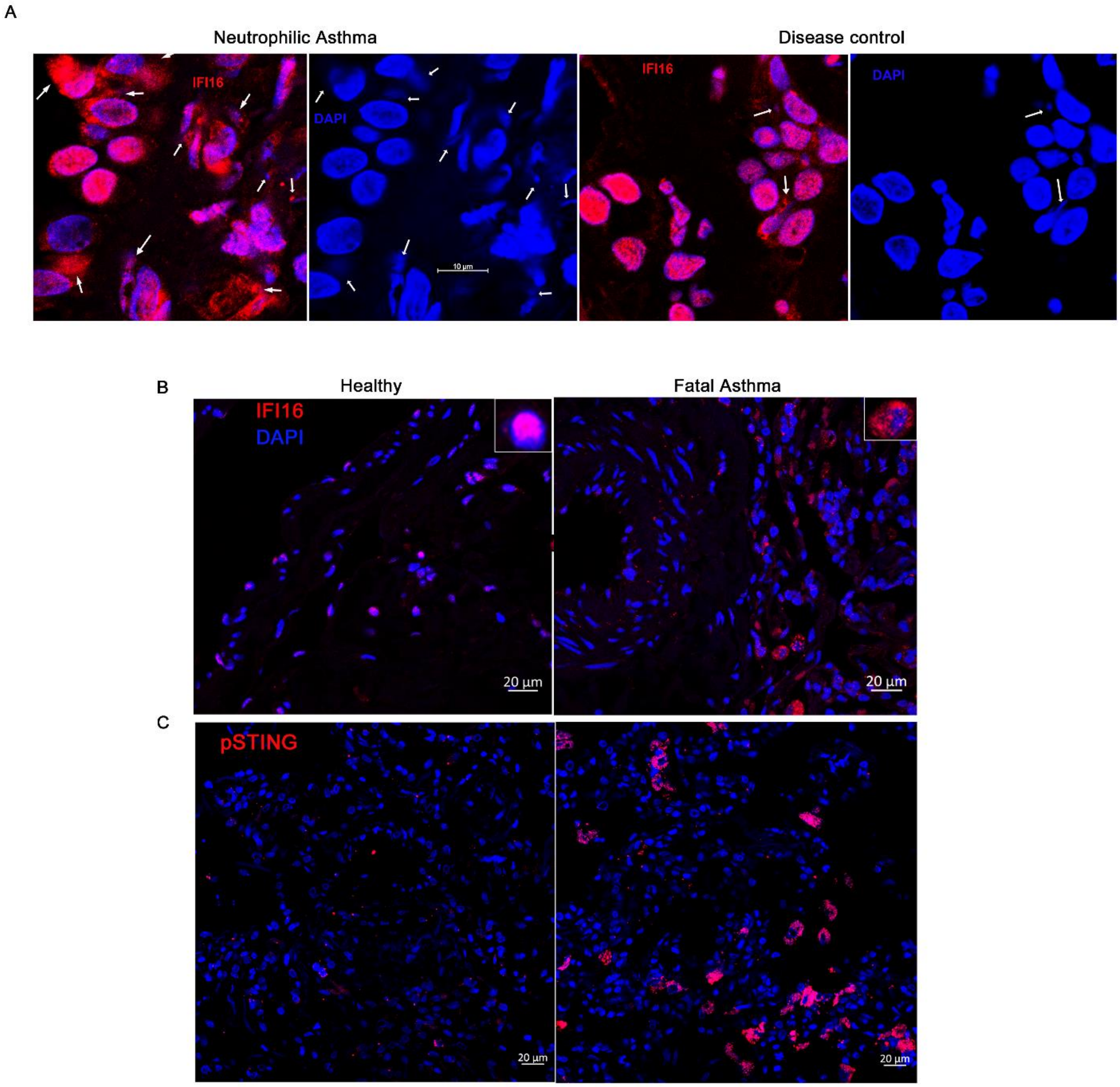


**Supplementary Fig. 7**. IFI16 and p-STING in the airway tissue from asthma patients. (**A**) Representative immunostaining of endobronchial biopsy specimens from a neutrophilic asthma patient and a disease control subjects for IFI16 (red). The tissue was counterstained with DAPI for DNA. Cytosolic IFI16 co-localizing with DAPI-staining extranuclear DNA is shown by white arrows (n=17 as per **Fig. 5A**). Scale bar, 10 μm. (**B and C**) Representative immunostaining of cadaveric lung sections from a healthy subject and an age- and gender-matched fatal asthma patient

for IFI16 and p-STING (red). Scale bar, 20 μm.

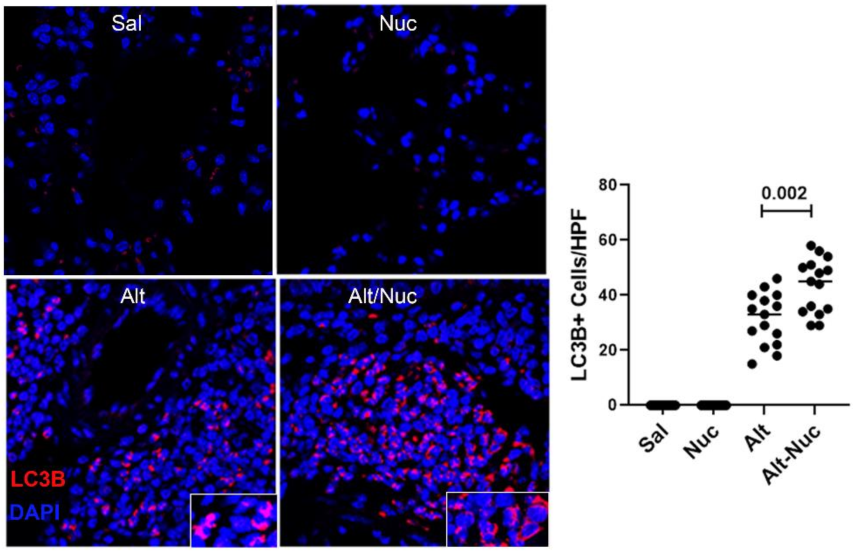


**Supplementary Fig. 8**. Autophagy in the asthma models. Representative immunostaining of lung tissue from Sal, Nuc, Alt and Alt-Nuc treated mice for the autophagy marker LC3B. The quantification of the LC3B+ cells from the 4 study groups is shown in the right panel. Scale bar, 20 μm.

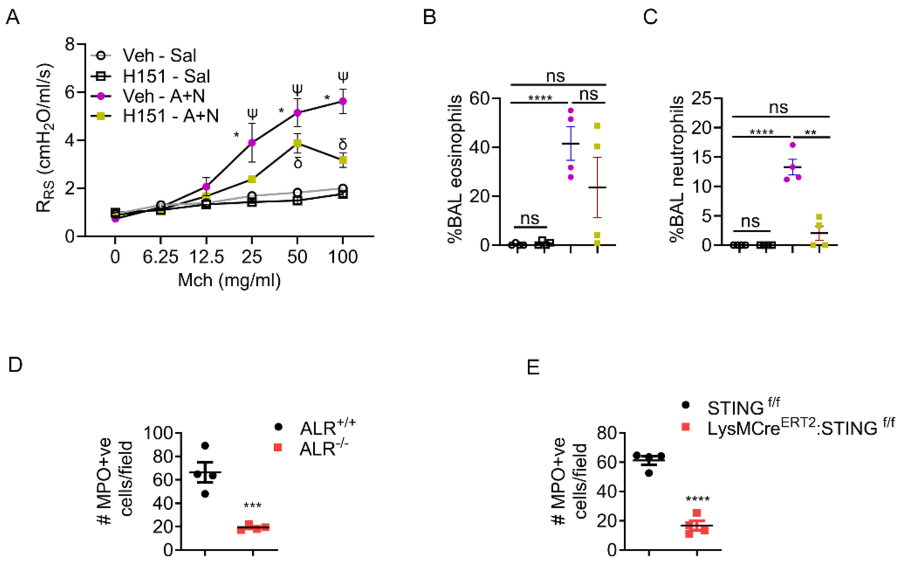


**Supplementary Fig. 9**. (**A-C**) B6 mice were pretreated i.p. with the STING inhibitor H151 (750 nmol H-151 per mouse in 200 µl 10% Tween-80 in PBS) or vehicle (10% Tween-80 in PBS) 1 hr before i.n. exposure to Alt-Nuc or Sal as per **Fig. 2A**. (A) AHR (lung resistance) in Veh-Sal, H151-Sal, Veh-A+N and H151-A+N groups as measured by flexiVent. $^{*}$(Veh-Sal vs Veh-A+N) *P*<0.0001; $^{\delta}$(Veh-Sal vs H151-A+N) *P*<0.0001; $^{\psi}$(Veh-A+N vs H151-A+N) *P*=0.006. (n=4; 2-way ANOVA, Tukey's multiple comparisons test). (**B and C**) Differential counts of BAL eosinophils and neutrophils. (**D and E**) The lung tissue from *ALR*$^{+/+}$, *ALR*$^{-/-}$, *Sting*$^{f/f}$ and *LysM*Cre:*Sting*$^{f/f}$ mice (n=4 per group) that were exposed to Alt-Nuc (as per **Fig. 2A**) was immunostained for neutrophil myeloperoxidase (MPO) and the MPO+ cells were quantified. Comparison made by Student's 2 tailed t-test. Data are presented as mean+/-SEM, **P*<0.05, ***P*<0.01, ****P*<0.001, *****P*<0.0001, ns=not significant.

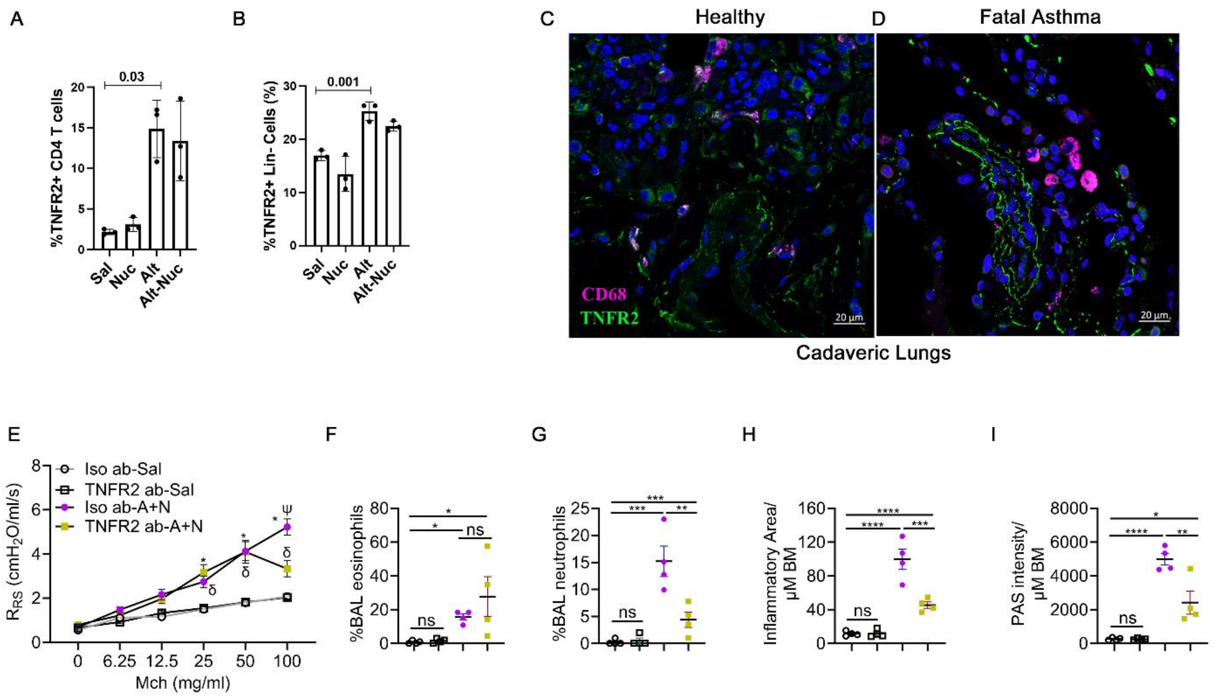


**Supplementary Fig. 10**. TNFR2 and asthma. ( **A and B**) TNFR2 induction by Alt. Isolated lung CD4+ and Lin- cells from the 4 mouse groups (Sal, Nuc, Alt, and Alt-Nuc) were analyzed by FCM (n=3 per group) ( **C and D**) Immunostaining for TNFR2 expressing blood vessels and cells, and CD68+ macrophages in cadaveric lungs from a healthy subject and a fatal asthma patient. Scale bar, 20 µm. ( **E-I**) Effect of an anti-TNFR2 antibody and an isotype control antibody. B6 mice were pretreated with 100µg of anti-TNFR2 antibody (clone TR75-54.7) or an isotype control antibody on day -1, +2 and +4 and then treated with Alt-Nuc or Sal as per **Fig. 2A**. (**E**) AHR (lung resistance) in Iso ab-Sal, TNFR2 ab-Sal, Iso ab-A+N and TNFR2 ab-A+N groups as measured by flexiVent. $^{*}$(Iso ab-Sal vs Iso ab-A+N) $P$<0.0001; $^{\delta}$(Iso ab-Sal vs TNFR2 ab-A+N) $P$<0.0001; $^{\psi}$(Iso ab-A+N vs TNFR2 ab-A+N) $P$<0.0001. (n=4; 2-way ANOVA, Tukey's multiple comparisons test). (**F-I**) Quantification of eosinophils and neutrophils in BAL, morphometric quantification of lung inflammation and mucus production in mice pretreated with Iso ab or TNFR2 ab and treated with Sal or A+N (Groups were color coded as in **E**). (n=4; 2-way ANOVA, Tukey's multiple

comparisons test). Data are presented as mean+/-SEM, *$P<0.05$, **$P<0.01$, ***$P<0.001$ ****$P<0.0001$, ns=not significant.

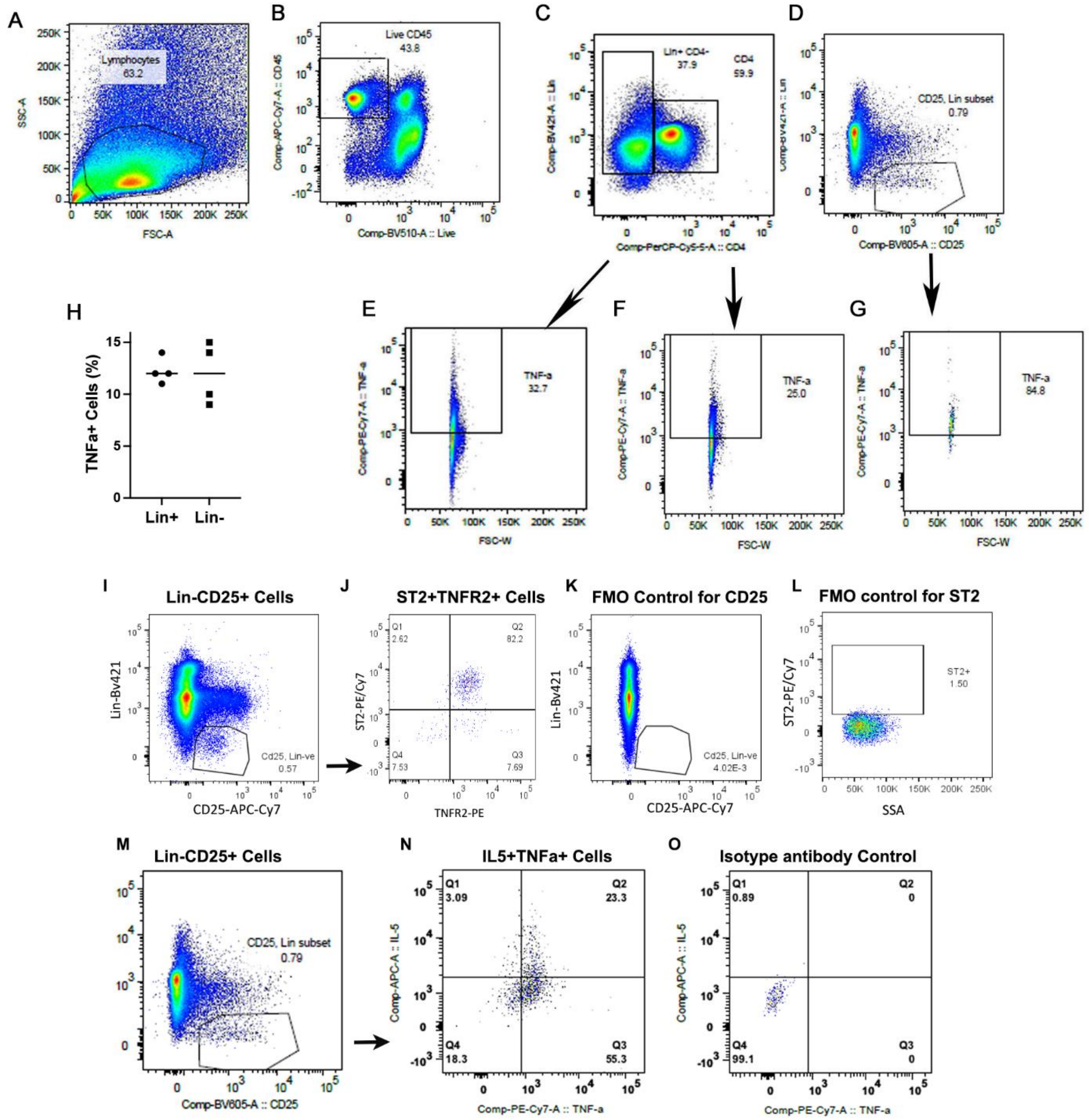


**Supplementary Fig. 11**. Flow cytometric analysis of TNFα+ lymphoid cells. **(A-G)** The flow cytometry gating strategy for detection of TNFα+ cells in Lin+CD4-, Lin+CD4+ and Lin-CD45+ cell populations in the lung cell digest. **(H)** Comparison of TNFα+ cells between Lin+ and Lin- CD45+ lung cells from Alt-treated mice. (**I-L**) Frequency of ST2+TNFR2+ ILC2s in Lin-CD25+ lung cell population and their FMO controls. (**M-O**) Frequency of IL5+TNFα+ cells

in lung ILCs (Lin-CD25+) and their isotype controls.
