# Supplementary material for: Allergens abrogate antiinflammatory DNA effects and unmask macrophage-driven neutrophilic asthma via ILC2/STING/TNF-α signaling
Source: J Clin Invest. 2025 Jun 17;135(16):e187907. doi: 10.1172/JCI187907 (PMC12352889; doi:10.1172/JCI187907)
Supplement: Unedited blot and gel images [file jci-135-187907-s048.pdf]

Uncropped blot images of Fig 4 I

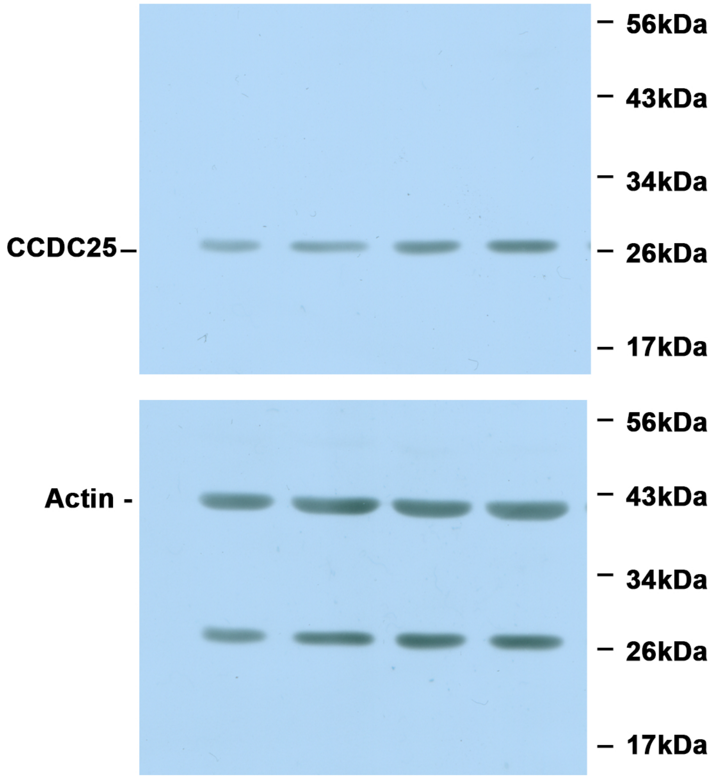

Uncropped blot images of Fig 4 J

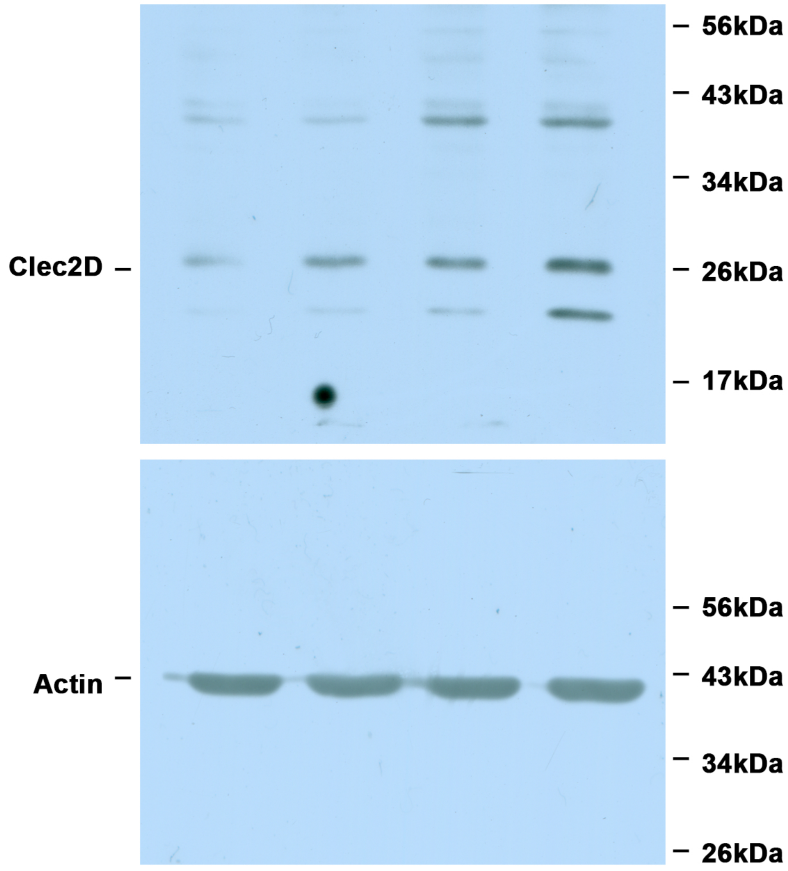

Uncropped blot images of Fig 4 K

Clec2D –

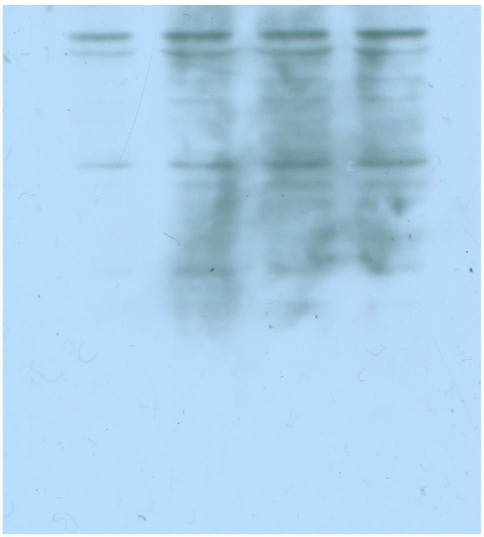

– 43kDa
– 34kDa
– 26kDa
– 17kDa

Actin

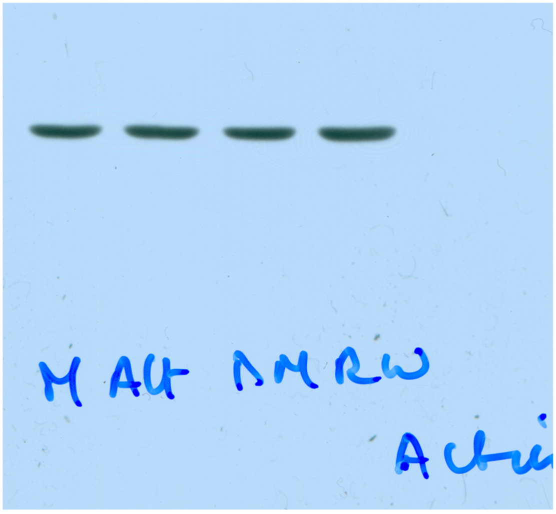

– 56kDa
– 43kDa
– 34kDa
– 26kDa
– 17kDa

Uncropped blot images of Fig 4 L

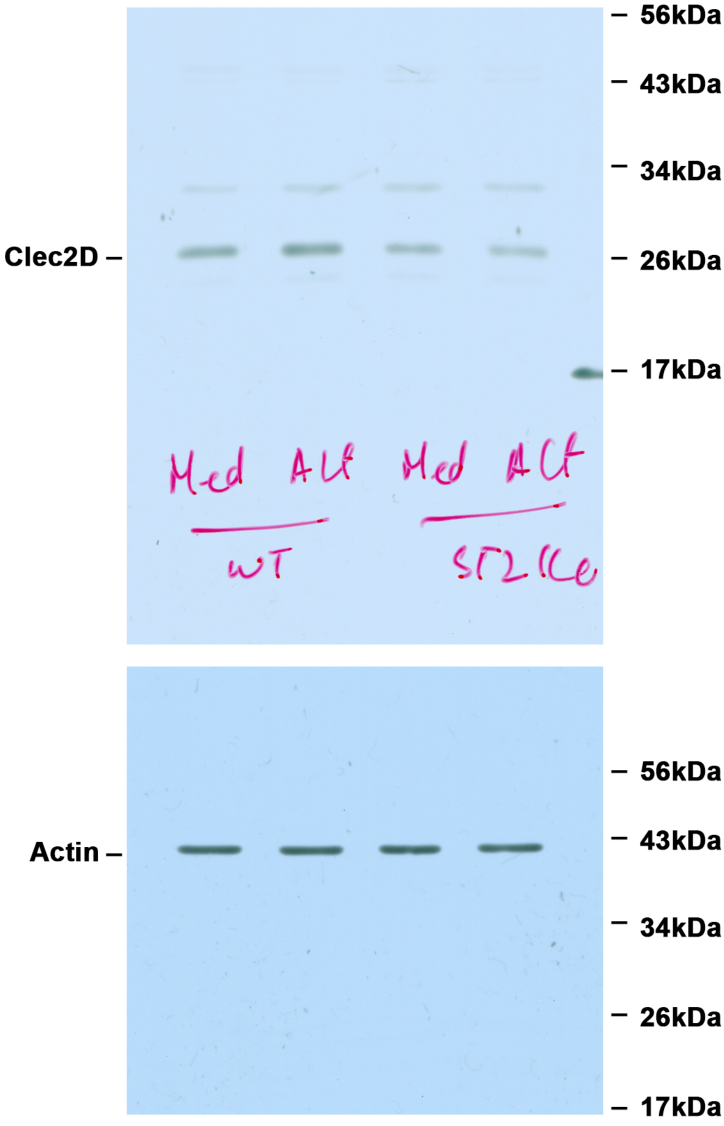

Uncropped blot images of Fig 4 N

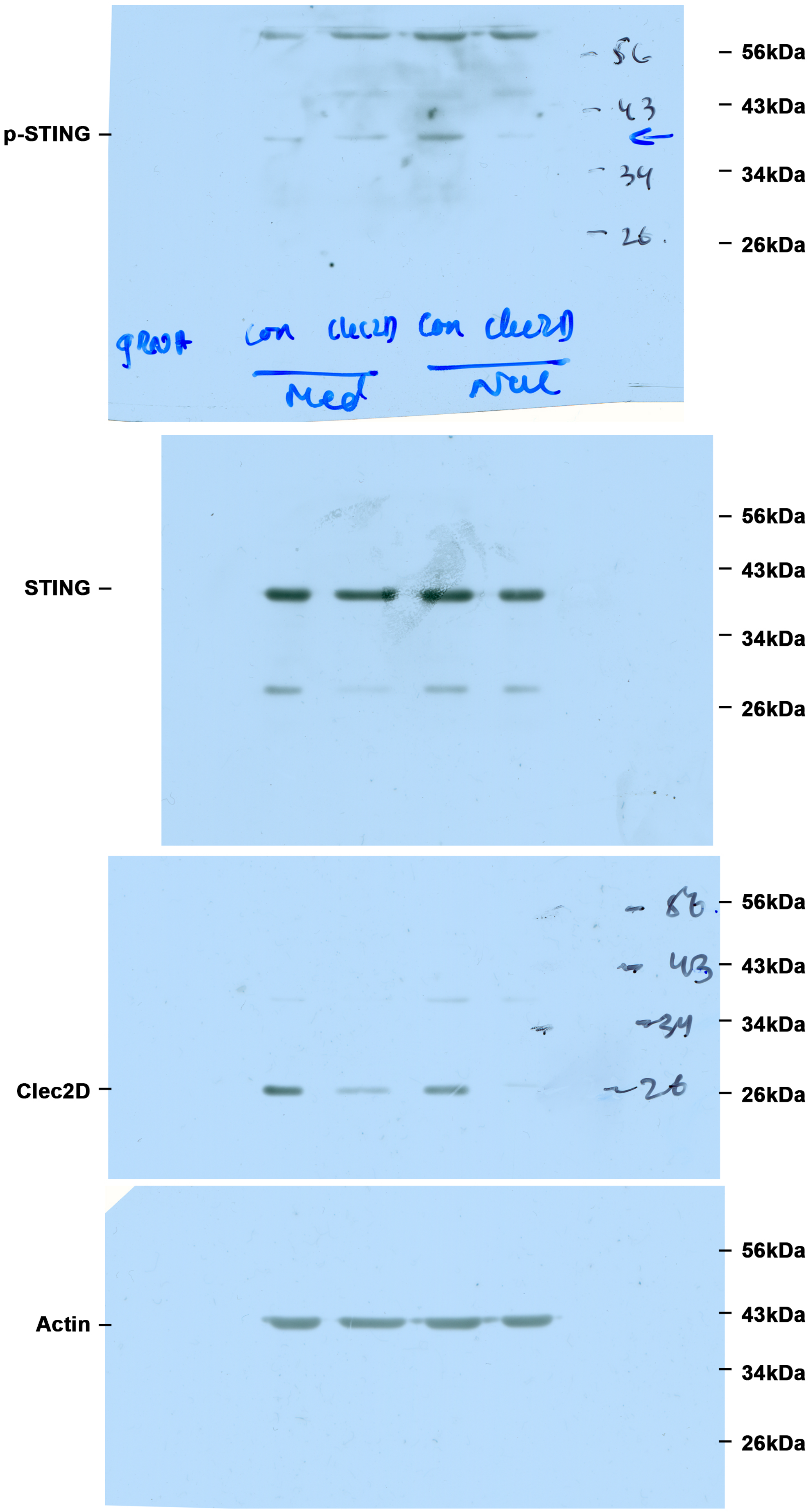

Uncropped blot images of Fig 5 E

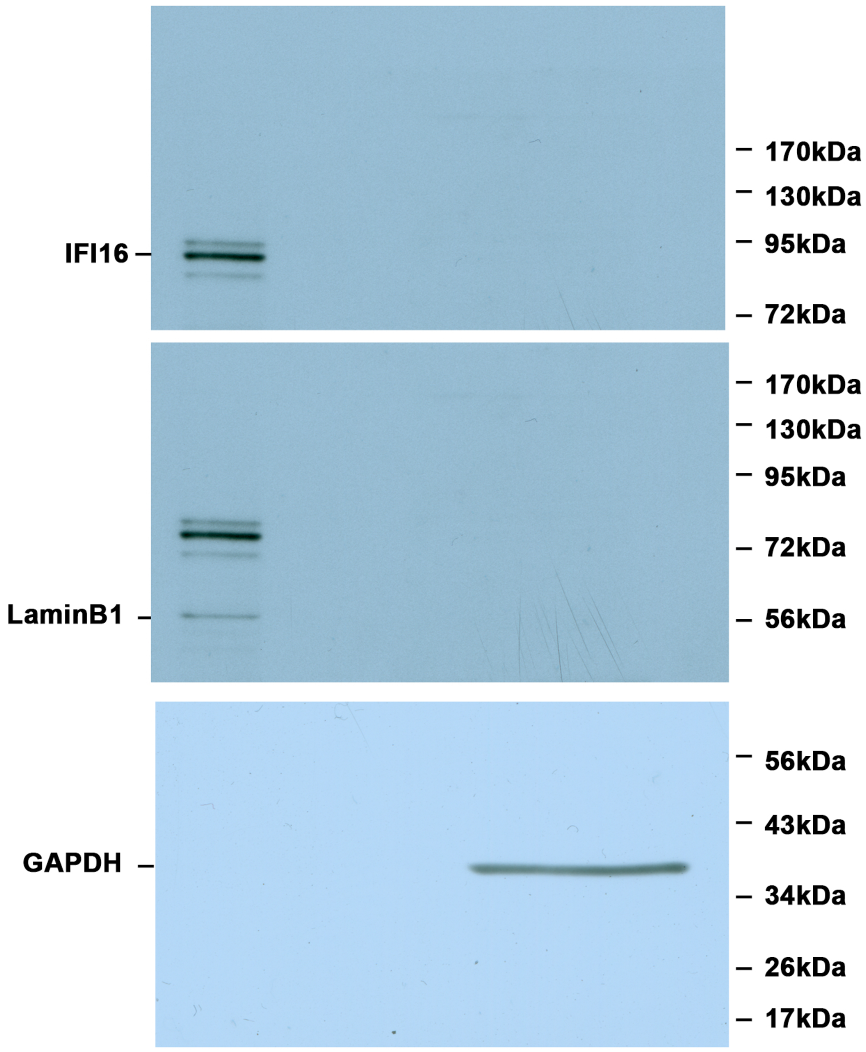

Uncropped blot images of Fig 5 F

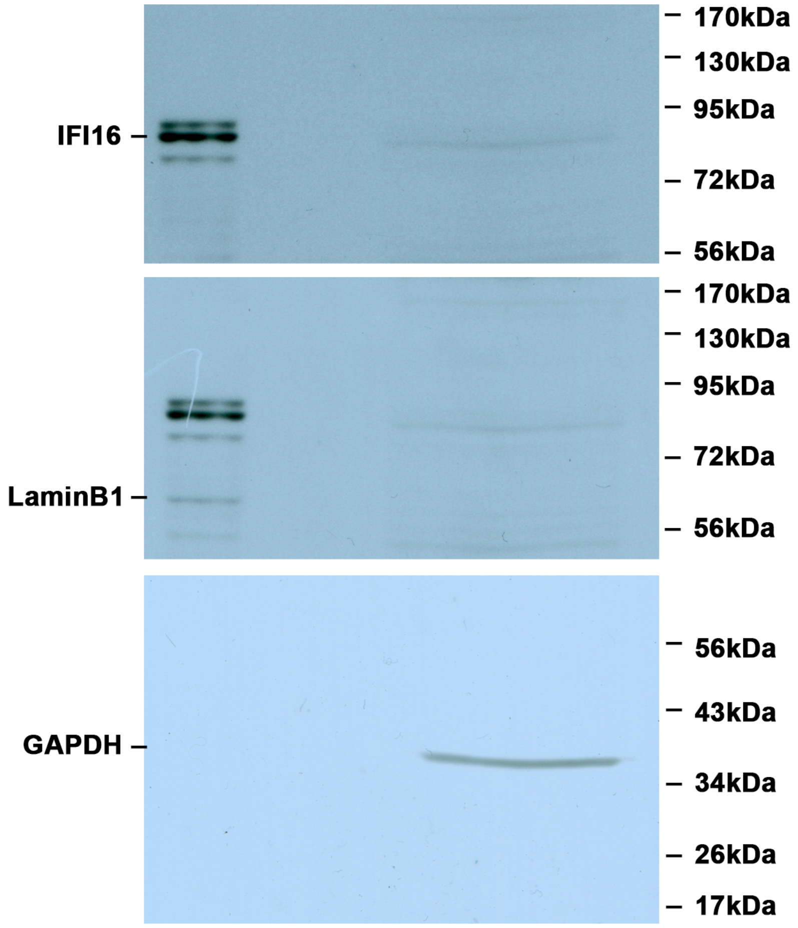

Uncropped blot images of Fig 5 I

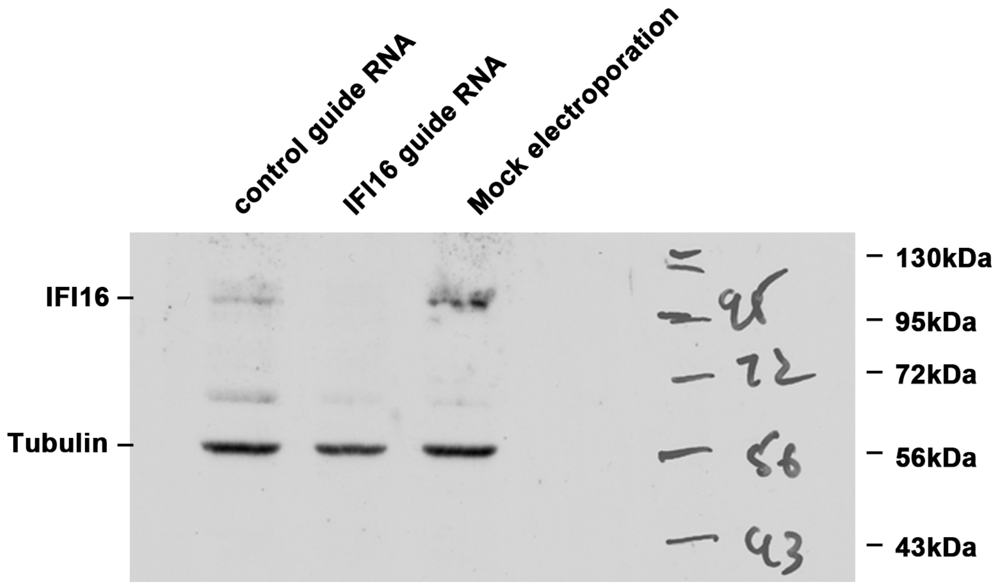

Uncropped blot images of Fig 6 B

pSTING

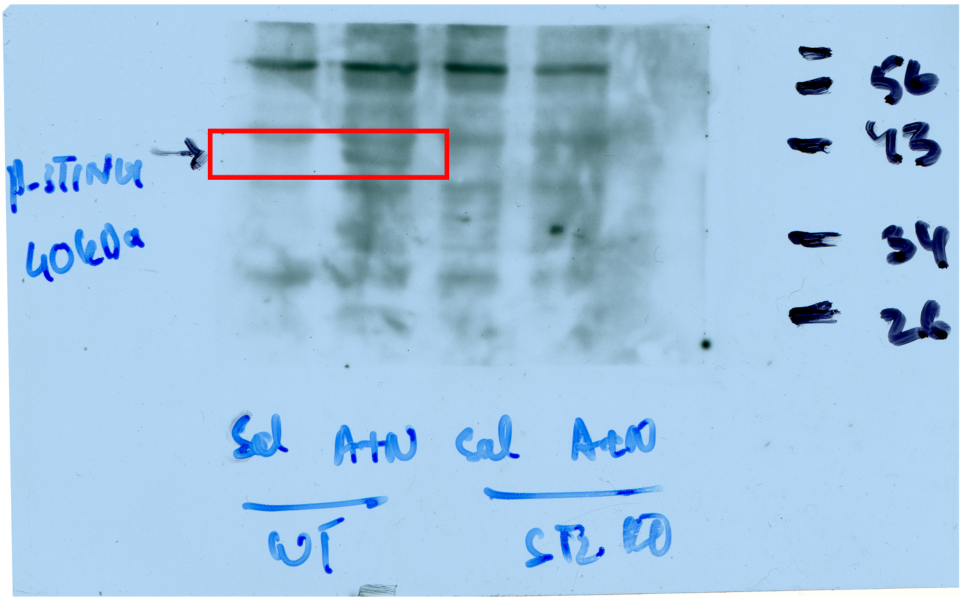

- 56kDa
- 43kDa
- 34kDa
- 26kDa

STING

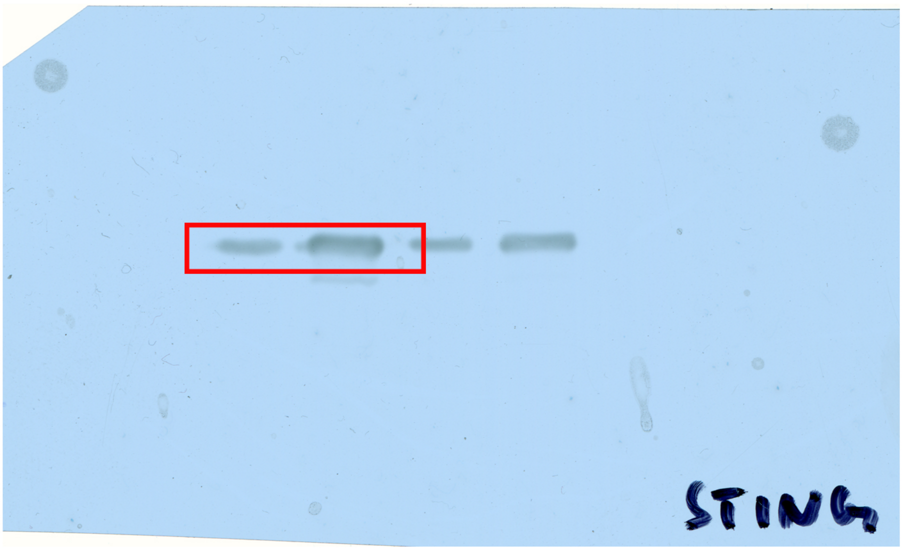

- 56kDa
- 43kDa
- 34kDa
- 26kDa

Actin

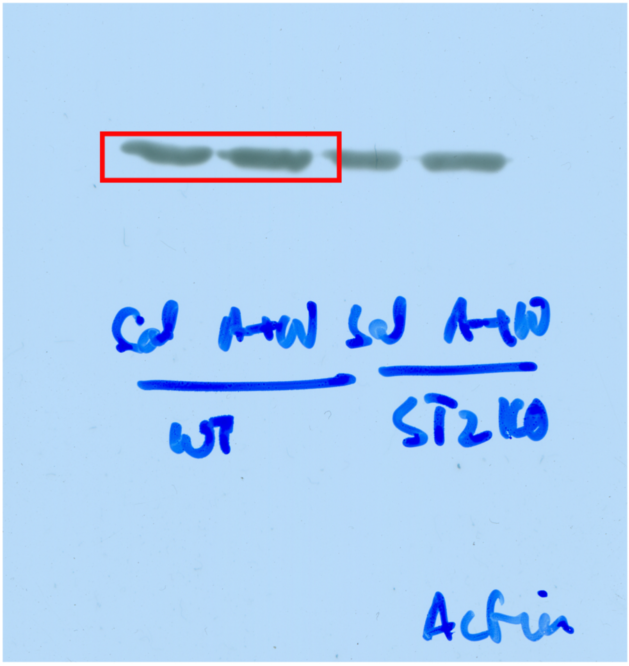

- 56kDa
- 43kDa
- 34kDa
